# Observations of the Outer Heliosphere, Heliosheath, and Interstellar Medium

**DOI:** 10.1007/s11214-022-00899-y

**Published:** 2022-05-31

**Authors:** J. D. Richardson, L. F. Burlaga, H. Elliott, W. S. Kurth, Y. D. Liu, R. von Steiger

**Affiliations:** 1 Kavli Institute for Astrophysics and Space Research and Department of Physics, Massachusetts Institute of Technology, Cambridge, MA USA; 2 NASA Goddard Space Flight Center, Code 673, Greenbelt, MD 20771 USA; 3 Department of Physics and Astronomy, University of Iowa, Iowa City, IA 52242 USA; 4 Southwest Research Institute, P.O. Drawer 28510, San Antonio, TX 78228 USA; 5 State Key Laboratory for Space Weather, Chinese Academy of Sciences, Beijing, China; 6 University of Chinese Academy of Sciences, Beijing, China; 7 Universität Bern, Bern, 2 Switzerland; 8 International Space Science Institute, Hallerstrasse 6, 3012 Bern, Switzerland

**Keywords:** Heliosphere, Solar wind, Interstellar medium, Heliosheath

## Abstract

The Voyager spacecraft have left the heliosphere and entered the interstellar medium, making the first observations of the termination shock, heliosheath, and heliopause. New Horizons is observing the solar wind in the outer heliosphere and making the first direct observations of solar wind pickup ions. This paper reviews the observations of the solar wind plasma and magnetic fields throughout the heliosphere and in the interstellar medium.

## Introduction

**Fig. 1:**
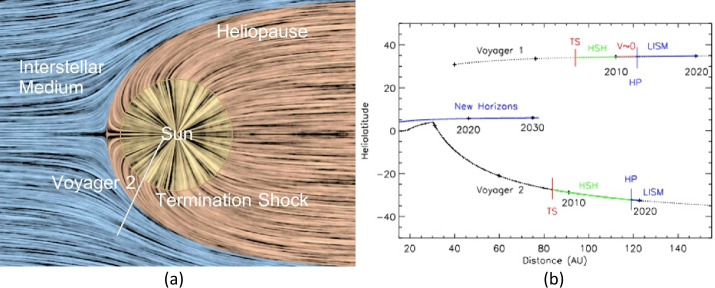
**a**) A schematic diagram of the interaction of the heliosphere and LISM looking down at the solar equatorial plane. **b**) The trajectories of V1, V2, and New Horizons and the locations of the V1 and V2 TS and HP crossings

The Sun moves through the local interstellar medium (LISM), the material between stars. Figure 1a shows a schematic of this interaction. Since the solar wind and LISM plasmas are both magnetized, they cannot mix, and a boundary forms between these plasmas called the heliopause (HP). This outer edge of the heliosphere was crossed by Voyager 1 (V1) at 121.7 AU and Voyager 2 (V2) at 119.0 Au. The solar wind ejected by the Sun is super fast-magnetosonic and thus a shock forms in front of the HP, the termination shock (TS). Voyager 1 (V1) observed the TS at 94 AU and Voyager 2 (V2) observed it at 84 AU. Between the TS and the HP is the heliosheath, a large region of shocked, compressed solar wind which is deflected away from the nose of the heliosphere and flows down the heliotail. The shape of the heliotail is controversial, with model predictions ranging from a spherical HP to a HP with a long comet-like tail (see Kleimann et al. 2022, this journal). Even inside these boundaries the LISM has a major influence on the solar wind. The LISM is about 2/3 neutral atoms and 1/3 plasma. The plasma cannot cross the HP, but the neutrals are unaffected by the magnetic fields and move into the heliosphere. These neutrals are ionized in the solar wind, forming pickup ions, which have an initial thermal energy equal to the solar wind flow energy (and much greater than the solar wind thermal plasma). These ions have a major effect on the solar wind inside the TS. The interactions between the solar wind and the LISM, and especially the observations of these regions and boundaries, are the foci of this chapter.

The outer heliosphere, beyond 10 AU, has been explored by 5 spacecraft, Pioneers 10 and 11, Voyagers 1 and 2, and New Horizons. Pioneer 10 went toward the heliotail, the others went toward the nose within 40° of the solar equatorial plane. Voyager 1 and 2 have provided the only data from the termination shock, the heliosheath, the heliopause, and the local interstellar medium (LISM). Power estimates indicate NH will also reach the TS and heliosheath (Stern et al. 2018). The influence of the LISM on the heliosphere was observed early in the Voyager mission. The first evidence of its effect was the inference that pickup ions provide the dominant pressure in pressure-balanced structures (Burlaga et al. 1994; Burlaga et al. 1996a,b). The next evidences were observations of the slowdown and heating of the solar wind by pickup ions (Richardson et al. 1995, 2008a; Elliott et al. 2016, 2019). The approach to the TS was signaled by leakage of energetic particles from the heliosheath into the heliosphere starting more than two years before the TS crossing. Near the TS, a slowdown of the solar wind was correlated with increased particle intensities (Florinski et al. 2009). The heliosheath was narrower than expected, turbulent, and marked by a steady average |B| and, at V2, by a fairly constant density N and speed |V|. Two years before V1 crossed the HP a decrease was observed in the radial speed V_R_ derived from particle data (Krimigis et al. 2019). The HP itself was a complex region with two significant jumps in the galactic cosmic rays (GCRs), dropouts of energetic electrons, and an increase in B, but surprisingly no change in the direction of B (Burlaga et al. 2013; Krimigis et al. 2013; Stone et al. 2013). A couple months after the HP crossing, plasma oscillations revealed a large increase in electron density indicative of LISM plasma (Gurnett et al. 2013). The LISM was discovered to be greatly affected by solar transients that drive shocks into the LISM that produce these plasma oscillations (Gurnett et al. 2013).

This chapter discusses the observations of these interactions by New Horizons, V1 and V2. We start with an overview of the heliosphere, then describe observations from inside the heliosphere outward into the LISM. Figure 1b shows the Voyager and New Horizon trajectories. Distances in AU are plotted versus heliolatitude and times are labeled on the trajectory traces. Also marked are the locations of the V1 and V2 TS crossings, the heliosheath (green), the quasi-stagnation region observed by V1 (red, marked V ∼ 0), the HP crossings, and the LISM (blue).

### The Plasma Heliosphere

**Fig. 2:**
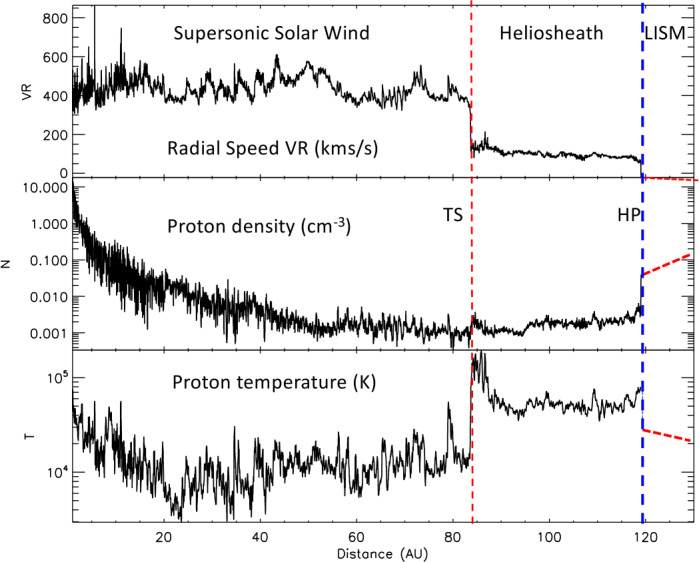
Plasma observations from 1 AU into the LISM

The Voyager spacecraft explored the heliosphere from Earth to the LISM, providing a snapshot of the heliospheric plasma, magnetic field, and particles. Figure 2 shows the radial plasma speed, density, and temperature from 1 AU into the LISM. Data inside the HP are 25-day running averages from the V2 plasma science (PLS) instrument and the densities outside the HP are from the V1 and V2 plasma wave subsystem (PWS). The radial speed varies between 300 and 850 km/s; variations smooth out at larger distances as the streams interact (Richardson and Burlaga 2013; Elliott et al. 2019).

At 20 AU, V2 observed solar minimum at low-latitudes with slow, dense cool solar wind. After solar minimum a roughly 1.3-year oscillation was observed at V2 and also at spacecraft throughout the heliosphere (Richardson et al. 1994; Gazis et al. 1995). A similar period was observed in cosmic rays, auroras, and convection speeds on the Sun. At about 50 AU, V2 observed the 2006 solar minimum when it was at 20° S heliolatitude. At these higher latitudes a significant fraction of fast wind was mixed in with the slower equatorial wind, giving a rise in speed to over 500 km/s and an increase in temperature.

Outside of 50 AU the solar wind is slowed down by pickup ions (PUIs); speeds are consistently below 400 km/s at both V2 and NH even though the solar cycle activity was quite different (Richardson et al. 1995; 2008a; Elliott et al. 2019). PUIs originate from LISM neutrals that are ionized and accelerated in the solar wind. The PUIs initially have thermal energies comparable to solar wind flow energies and by 15 AU dominate the plasma thermal pressure. The PUI fraction was ∼17% at the TS giving a 18% slowdown of the solar wind, so roughly 1/3 of the solar wind flow energy is transferred to the PUIs before the TS.

The solar wind and heliosphere vary with the solar cycle. At solar minimum the Sun’s dipole tilt is small and the coronal holes accelerate solar wind at high-latitudes. Corotating interaction regions (CIRs) form when high speed solar wind streams overtake slower streams, with a forward-reverse shock pair forming at the interface; these are most prevalent in the declining phase of the solar cycle. The largest solar transients are coronal mass ejections (CMEs), which propagate through the heliosphere as interplanetary CMEs (ICMEs). ICMEs expand outward to about 15 AU until they reach equilibrium with the surrounding solar wind (Richardson et al. 2012). ICMEs propagate through the heliosphere to the HP, where they can drive shocks in the VLISM (Kim et al. 2017; Liu et al. 2014; Gurnett et al. 2013). More ICMEs occur at solar maximum. These ICMEs merge, with slow ICMEs overtaking fast ones, to form merged interaction regions (MIRs). Shocks driven by ICMEs are observed in the solar wind out to the TS, with the last large shock, observed at 78 AU (Richardson et al. 2008a), showing jumps in B, V_R_, density N, a large increase in the temperature T, and enhanced energetic particle fluxes.

The TS is described in detail below; at the TS the speed drops by a factor of 2, the density goes up by a similar factor, and T increases by an order of magnitude (Richardson et al. 2008b). V_R_ drops slowly across the heliosheath. N decreased after the TS at solar minimum, then increased at ∼105 AU when solar maximum reaches the heliosheath, then was fairly constant until the HP. T decreased from 120,000 °K after the TS to 50,000 °K outside ∼90 AU. The MIRs driven by ICMEs are still observed in the heliosheath (see section below) with two examples at ∼100 and ∼109 AU. Outside the HP, the Voyager PWS instruments determine the plasma density when plasma oscillations are observed (Gurnett and Kurth 2019; Gurnett et al. 2021). These densities increase away from the HP. PLS data suggest the plasma T is high, 30,000 °-50,000 °K, after the HP (Richardson et al. 2019); T must eventually decline to the LISM value of 6400 °K (Swaczyna et al. 2021).

### The Energetic Particle Heliosphere

**Fig. 3:**
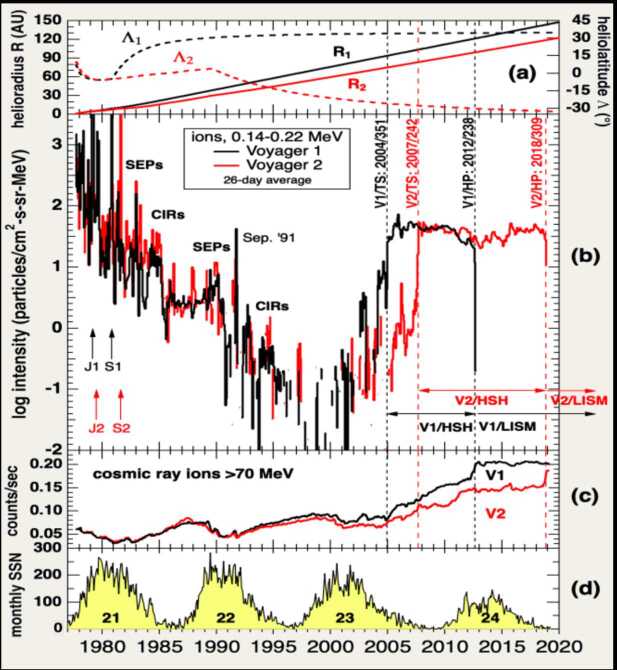
Particle observations from 1 AU into the LISM (Dialynas et al. 2022). (**a**): Distance and heliolatitude of V1 (white) and V2 (blue). (**b**): 140-220 keV ions from V1 (white) and V2 (blue). (**c**): >70 MeV cosmic rays from V1 (white) and V2 (blue). (**d**): Sunspot number

In addition to the plasma heliosphere, Voyager has also observed the energetic particle heliosphere. Figure 3 shows the distance and heliolatitude of V1 and V2 in panel (a), 140-220 keV ions in panel (b), >70 MeV GCRs in panel (c), and the sunspot number in panel (d). The keV particles peak near the Sun, where they are accelerated by shocks driven by ICMEs or corotating interaction regions (CIRs). Their intensities increase with distance, with occasional increases at large events such as the GMIR in Sept 1991. At 65-75 AU the intensities reach a minimum. Then, almost 10 AU before the TS crossing, the intensities increased due to particles accelerated at the TS leaking inwards into the supersonic wind. These particles are observed when field lines connect the TS and the spacecraft; the intensities increase up to the TS.

At the TS, where these particles are accelerated, the intensity jumps, then remains fairly steady in the heliosheath with a slow decrease as the spacecraft approach the HP. Note that even though V1 and V2 are separated by over 100 AU, the intensities are very similar. At the HP the intensities rapidly decrease to background levels (see HP section). The GCRs come from outside the heliosphere and their intensities generally increase with distance. They are modulated by the solar cycle, with less at solar maxima when magnetic fields are higher. At the TS the slope increases, then the intensity takes a final jump at the HP (for more details see Rankin et al. 2022).

## The Solar Wind in the Outer Heliosphere

Many features of the evolution of the solar wind are discussed in previous reviews (Burlaga et al. 1996; Richardson and Burlaga 2013; Richardson and Stone 2009; von Steiger and Richardson 2006; Burlaga et al. 2021b); therefore, we provide only a high-level overview. New Horizons (NH) provides the most recent solar wind data from the outer heliosphere. NH does not have a magnetometer; however, the plasma instrument provides excellent H^+^ and He^++^ data and the first pickup ion measurements from the outer heliosphere.

**Fig. 4:**
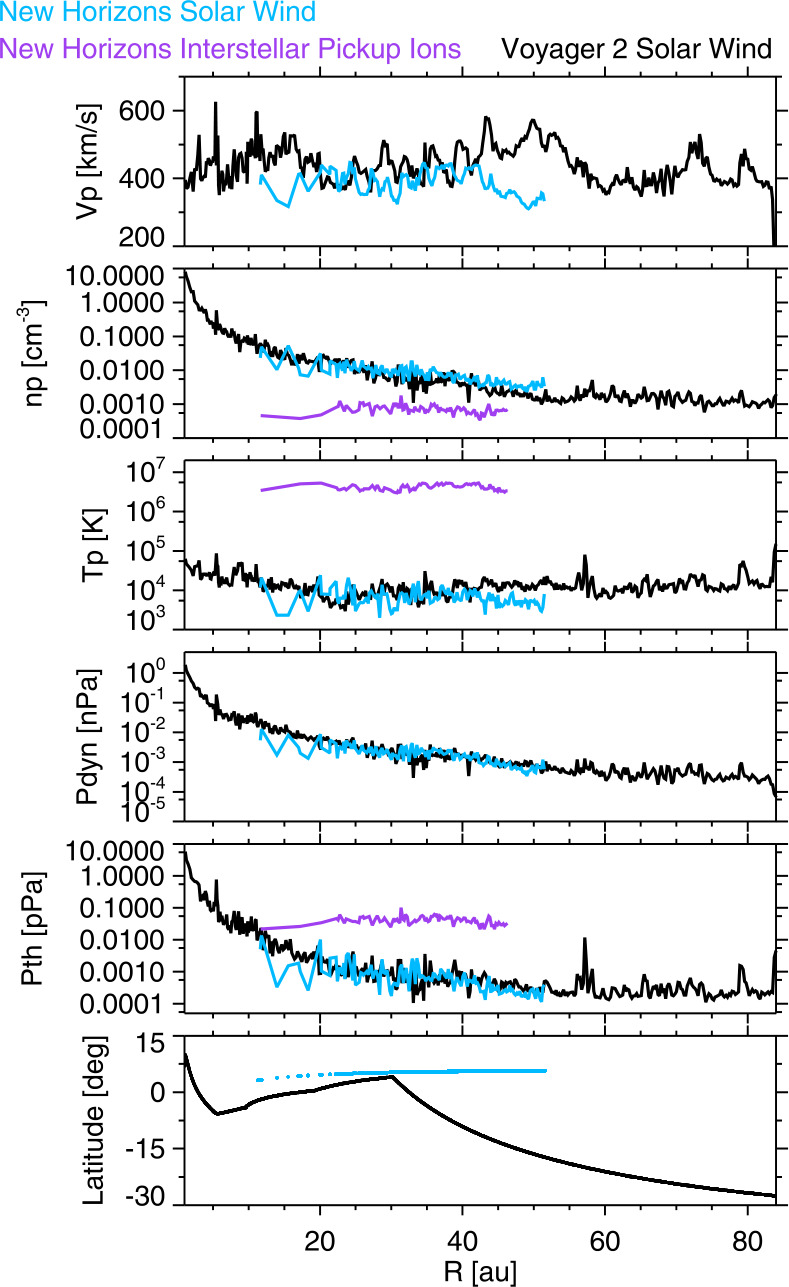
Solar rotation averages of V2 and NH H^+^ speeds, densities, temperatures, dynamic pressure, thermal pressure, and heliolatitude. The NH thermal (blue) and PUI (purple) densities and temperatures are both shown

Figure 4 shows the V2 and NH solar rotation averaged plasma speeds, densities, temperatures, dynamic pressures, and thermal pressures versus distance. The bottom panel shows the spacecraft heliolatitudes. NH samples distances observed by V2 about 25 years earlier. NH remains at low heliolatitudes whereas V2 moved south after the Neptune encounter at 30 AU. NH speeds are close to, but slightly below the V2 speeds. The difference grows larger outside ∼43 AU; this increase is probably because V2 observed solar minimum conditions at these distances and, since it was at 20° S, it observed a mixture of fast and slow solar wind. Both spacecraft observed an R^−2^ density fall-off as expected. The NH densities are slightly higher than those at V2 inside 43 AU and significantly higher outside 43 AU, giving similar fluxes. The NH interstellar pickup ion (PUI) densities shown in purple are fairly constant, so the percentage of PUIs increases with distance; at 50 AU about 8% of the ions are PUIs (McComas et al. 2017, 2021). The solar wind thermal ion temperatures decrease to 20-25 AU, then increase due to energy transfer from the PUIs (Smith et al. 2006). The NH PUI temperatures are fairly constant from 10-46 AU at near $4\times 10^{6}$ K; the PUIs are also heated as they move outward (see Zirnstein et al. 2022). The NH thermal pressure plot shows that the PUI pressure dominates the thermal pressure outside ∼12 AU. The physics of the plasma changes when this occurs, with wave speeds determined by the PUIs and shocks preferentially heating the PUIs.

### Interstellar Pickup Ion Effects

The first observational evidence for interstellar PUIs were observations of pressure balance structures in the solar wind; outside of 20 AU a hot PUI component was required if the structures were in pressure equilibrium (Burlaga et al. 1994). The increase in temperature outside 20-25 AU discussed above is another effect of the PUIs; the initial PUI ring distributions are unstable and drive magnetic waves which heat the thermal plasma (Smith et al. 2006; Isenberg et al. 2005). The energy to heat the PUIs comes from the solar wind flow energy; slowing of the solar wind is first observed at about 30 AU (Richardson et al. 1995; Wang and Richardson 2003; Elliott et al. 2019) and the slowdown is about 17% ahead of the TS (Richardson et al. 2008a). The solar wind slowdown is related to the percentage of PUIs by n_pui_/n_sw_ = ($3\gamma - 1)/ (2\gamma -1) (\delta \text{v/v}_{0}$), where n_pui_ is the PUI density, n_sw_ is the thermal solar wind proton density, $\delta $v is the solar wind speed decrease, v_0_ is the unperturbed solar wind velocity, and $\gamma $ is the ratio of specific heats (Lee 1995; Richardson et al. 1995). For $\gamma = 5/3$, n_pui_/n_sw_$= 7/6 (\delta \text{v/v}_{0})$ and a 17% slowdown implies that PUIs make up 20% of the solar wind (although we note below that $\gamma $ may not be constant).

**Fig. 5:**
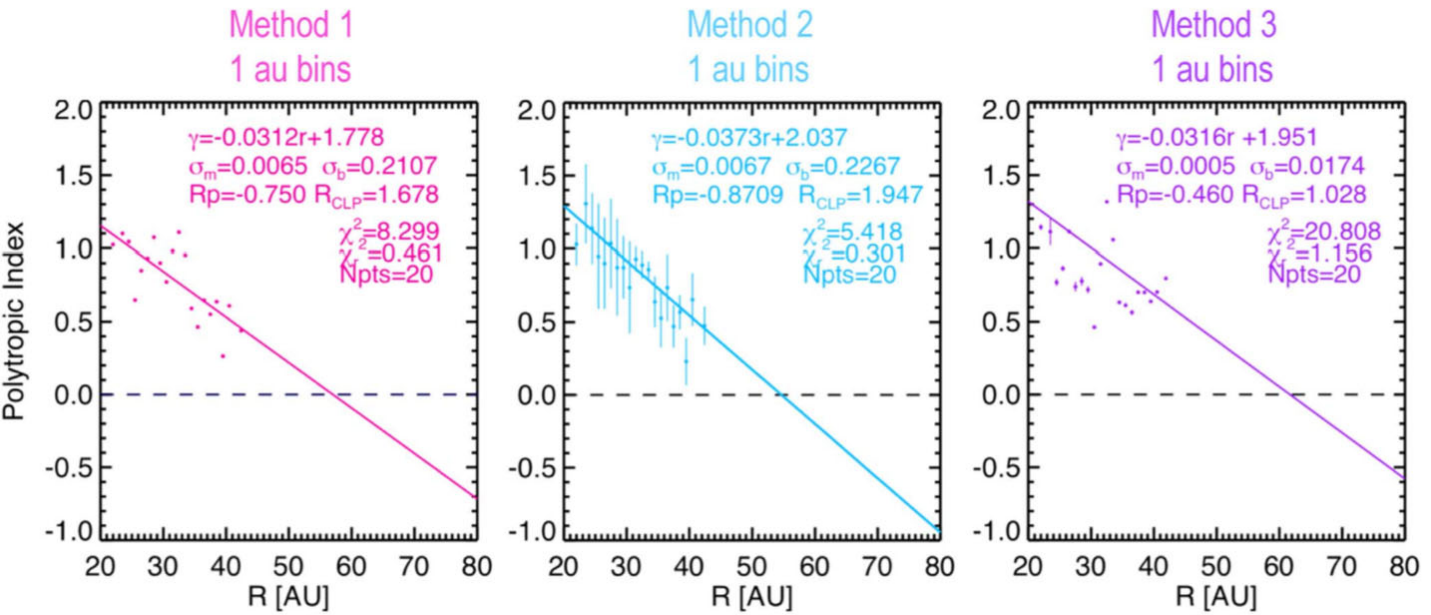
Plots of the solar wind polytropic index as a function of distance determined using methods 1-3 described in Elliott et al. (2019) (Fig. 10 from Elliott et al. 2019)

Elliott et al. (2019) tested the Richardson et al. (1995) equation by using pickup ion and solar wind densities measured with solar wind to calculate n_pui_/n_sw_ and using the speed at NH and speed measurements at 1 AU from ACE and STEREO to estimate the amount of slowing ($\delta $v/v_0_). They found that when they used a constant polytropic index the two sides of the Richardson et al. 1995 equation did equate. With investigation it became clear that the relationship between the solar wind density and solar wind temperature slowly and systematically evolved with distance. Elliott et al. (2019) fit plots of solar wind density and temperature measurements for given distance ranges using 3 methods. Method 1 fit a simple scatter plot with a line for a given distance range. Method 2 binned the temperature into density bins for a given distance range and fit the binned results. Method 3 fit sets of 8 adjacent points and then binned the polytropic index results by speed for a given distance and determined an index since the index did not vary much by speed. All 3 methods produced a solar wind polytropic index that decreased with increasing distance (Fig. 5). Theoretically, the polytropic index usually refers to the entire plasma and not only the solar wind portion. However, the physical effect encapsulated by the radially decreasing polytropic index determined for the solar wind portion of the data is that the solar wind temperature and density relationship is slowly evolving with increasing distance as the addition of interstellar pickup ions heats and slows the solar wind.

**Fig. 6:**
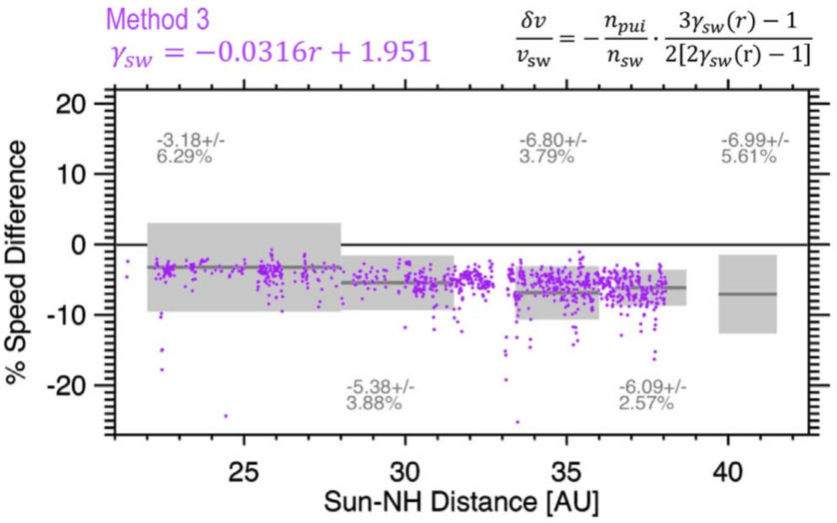
The average measured solar wind slowing with standard deviations (solid grey line and grey bar) and, in purple, the estimated amount of slowing determined from the NH measured n_pui_/n_sw_ in the solar wind using the Richardson et al. (1995) formula, and the method 3 solar wind polytropic index radial variation with distance determined from solar wind observations (Fig. 15 from Elliott et al. 2019)

When Elliott et al. (2019) substituted the formula for the polytropic index as a function of distance into the formula derived by Richardson et al. (1995), both sides of the equation agreed and the measured slowing was consistent with the measured PUI density (Fig. 6). Additional measurements are required to quantify how the solar wind is heated and slowed by picking up interstellar material. One needs to measure solar wind parcels as they move away from the Sun, the interstellar neutrals, and the pickup ions simultaneously. Although such measurements do not exist, the interaction between the solar wind and interstellar material can be simulated in a more self-consistent way than is done in the work of Richardson et al. (1995) and Elliott et al. (2019) in order to better understand how the solar wind is heated and slowed as interstellar material is picked up by the solar wind on its journey through the heliosphere.

### Solar Wind Magnetic Fields Between 7 and 87 AU

**Fig. 7:**
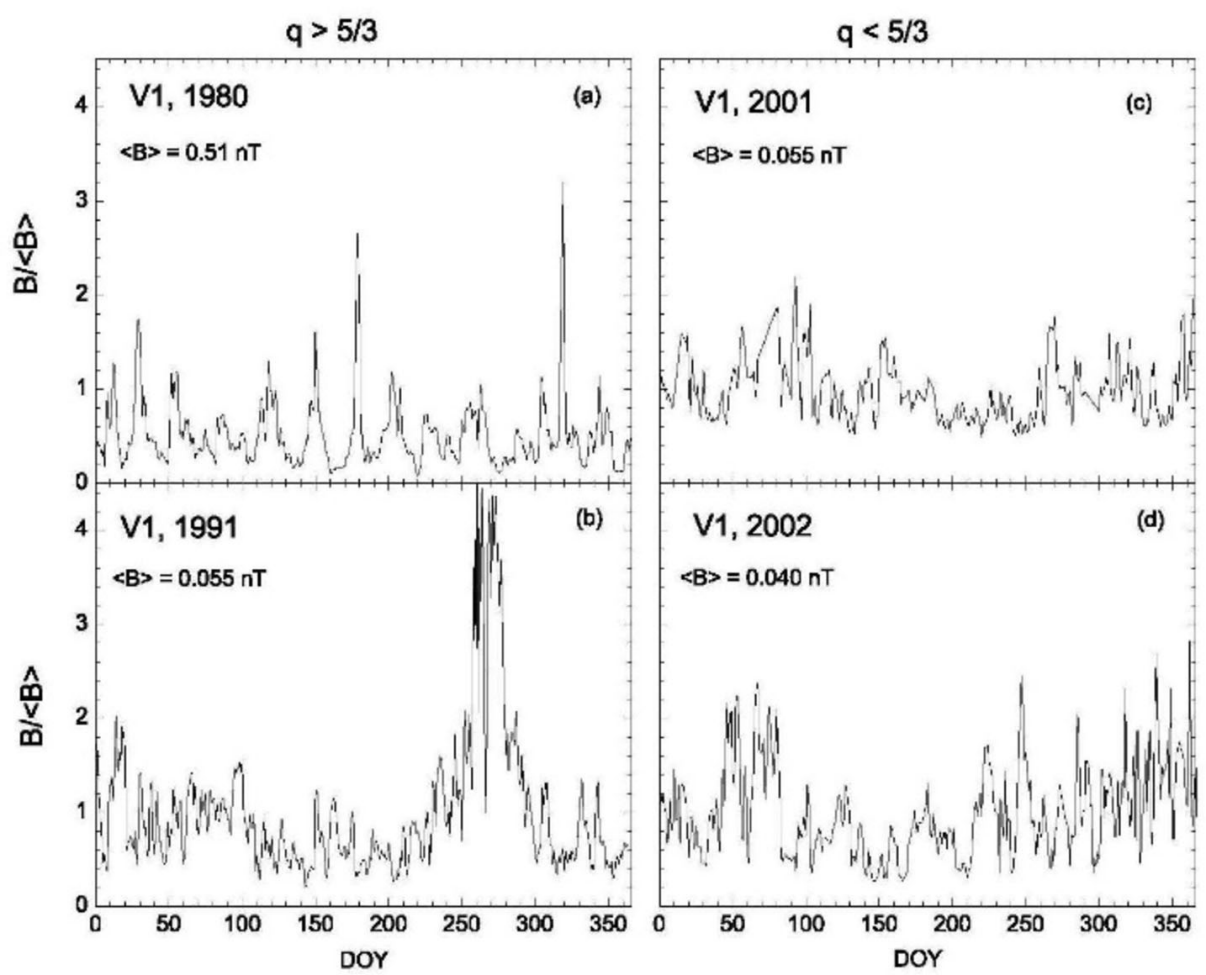
The V1 B/〈B〉 in four different years

This section describes observations of B in the solar wind by Voyager 1 between 7 AU and 80 AU on scales from one to 128 days (Burlaga and Vinas 2005a). Relatively large clusters of strong magnetic fields, MIRs, move past a spacecraft during a fraction of a solar rotation. MIRs form near one AU and can persist through the heliosheath to the heliopause. During the declining phase of the solar cycle, MIRs often occur quasi-periodically, as shown in data from 1980 in Fig. 7(a). More commonly, MIRs occur sporadically, as illustrated in Fig. 7(d) (Burlaga and Vinas 2005a). MIRs can merge to form larger clusters of strong magnetic fields, called global merged interaction regions, GMIRs, that encircle the sun and extend to high latitudes. GMIRs typically form near ∼20-30 AU, and persist throughout the heliosphere and heliosheath as shown in Fig. 1(b).

All of these interaction regions have “filamentary structures” associated with jumps in B of various sizes over a wide range of scales. Jumps in B and filamentary structures, seen in all of the panels in Fig. 7, are fundamental features of the heliosphere and heliosheath, related to the fact that the solar wind is a driven, non-linear, non-equilibrium system. These features are characteristic of the multifractal structure of the solar wind and heliosheath magnetic field (Burlaga 1995, 2001, 2004).

Boltzmann-Gibbs statistical mechanics, with an additive entropy function, cannot describe non-equilibrium physical systems with large variability and multifractal structure. Tsallis (1988, 1994, 2004a) introduced a generalization of Boltzmann-Gibbs statistical mechanics in which the entropy is non-additive (non-extensive) and he introduced a probability distribution function, the Tsallis distribution function, that can describe observations of the magnetic field in the heliosphere and heliosheath. The Tsallis distribution is proportional to the q-exponential function $\exp_{\mathrm{q}} (- \text{B}_{\mathrm{q}} \text{E}) = (1 - (\text{q} - 1) \text{B}_{\mathrm{q}} \text{E})^{-1/(\text{q} - 1)}$. When q = 1 the q-exponential function is a Gaussian distribution. The q-exponential function approaches the form of an exponential function for small E and a power law function for large E. The 2nd moment of the q-exponential function is finite for q < 5/3 and diverges for 5/3 ≤ q ≤ 3. The kappa function, which has been used in plasma physics to model speed distribution functions (Olbert 1968; Vasyliunas 1968), is the q-exponential distribution with q = 1 + 1/$\kappa $.

One can quantitatively describe the jumps and filamentary structure by studying the normalized increments of B, dBn(t_i_) = (B(t_i_) + t_n_) - B(t_i_))/〈B(t_i_)〉, on scales t_n_=$2^{\mathrm{n}}$ days, where n = 0, 1, 2, 3, 4, 5, 6, and 7 and t_i_ is time. We consider scales from 1 to 128 days. The distribution of increments of dBn(t_i_) is described by the Tsallis distribution R_q_(x) = A(1 + (q − 1) B_q_(dBn)^2^ ^(−1/(q − 1))^, which is the fundamental distribution function in non-equilibrium statistical mechanics (Tsallis 1988). The non-extensivity parameter q for a Gaussian distribution is q = 1, and in general q > 1.

**Fig. 8:**
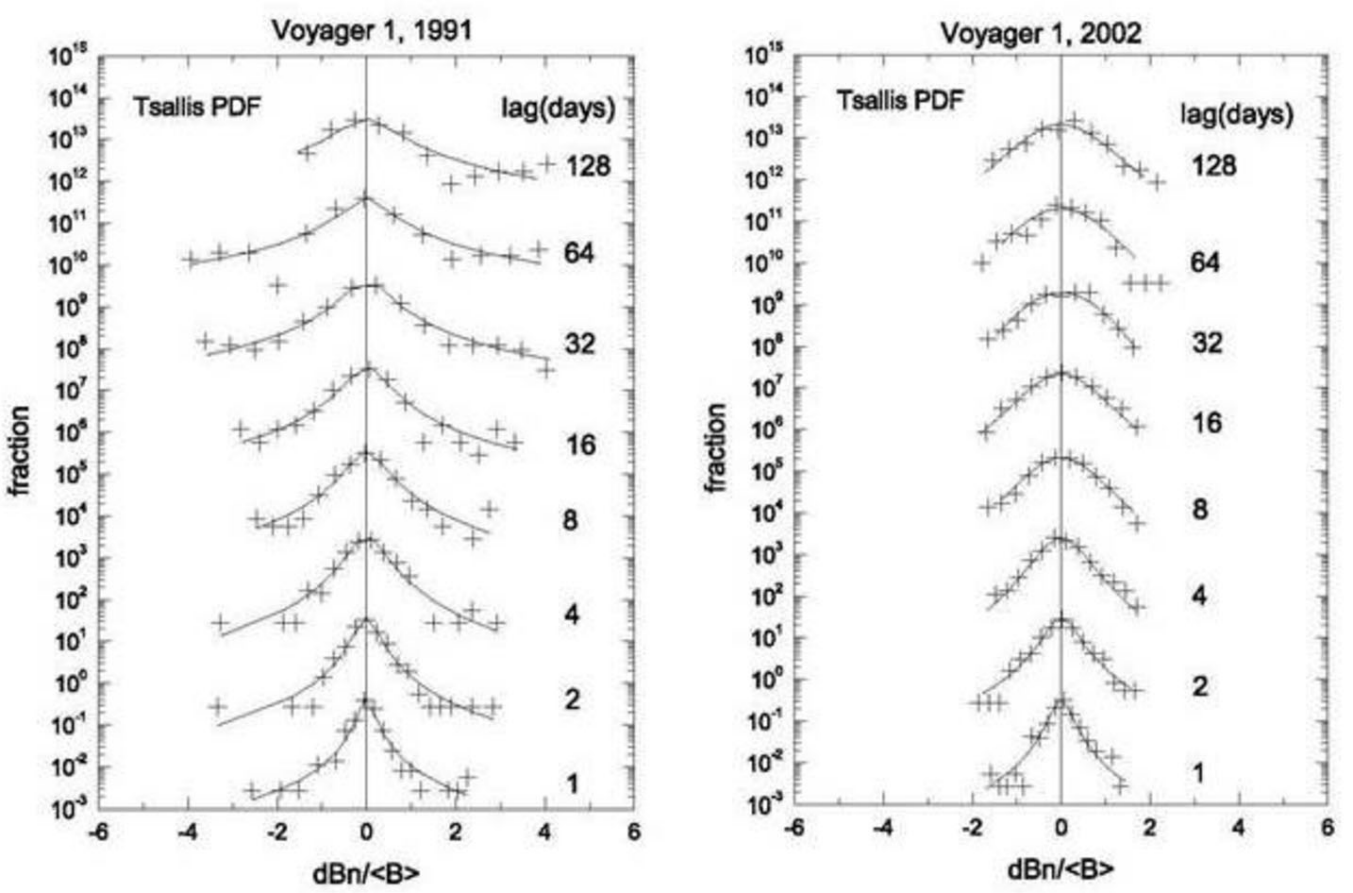
Distribution of the increments dBn(t_i_) (shown by the plus signs) for a given lag in units of days and fits using the Tsallis distribution

Figure 8 shows the distribution of the increments dBn(t_i_) (shown by the plus signs) for a given lag in units of days, ranging from one day to 128 days, as shown on the right side of each panel. Each distribution function was fit with the Tsallis distribution shown as solid curves for each lag t$_{\mathrm{n}} = 2^{\mathrm{n}}$ days, where n = 0, 1, 2, 3, 4, 5, 6, and 7.

During 1991, Voyager 1 was in the supersonic solar wind at 45 AU. The observed distributions of increments of B (shown by the plus signs) were highly non-Gaussian. (A Gaussian curve would appear as a parabola on the log scale in Fig. 8). This result clearly demonstrates that the large-scale magnetic fluctuations of B in the supersonic wind cannot be described by Boltzmann statistics. But the distributions can be described by the Tsallis distribution of non-extensive statistical mechanics, which are shown by the solid curves. The column on the right in Fig. 8 shows observations made by Voyager 1 during 2002 near 87 AU, 7 AU before the termination shock crossing. Non-Gaussian (q > 1) Tsallis distributions are observed on scales from 1 to at least 4 days, but at larger scales the distributions are Gaussian.

Figure 8 shows data from the CIRs in panel (a) and from a GMIR in panel (b) of Fig. 7. The very large jumps in B/〈B〉 are the cause of the non-Gaussian behavior. This behavior is common near 20 - 25 AU. However, at larger distances (during 2002 for example) jumps in B were present only at relatively small scales, as expressed by the non-Gaussian fits with a Tsallis distribution on scales from 1 to 4 days.

**Fig. 9:**
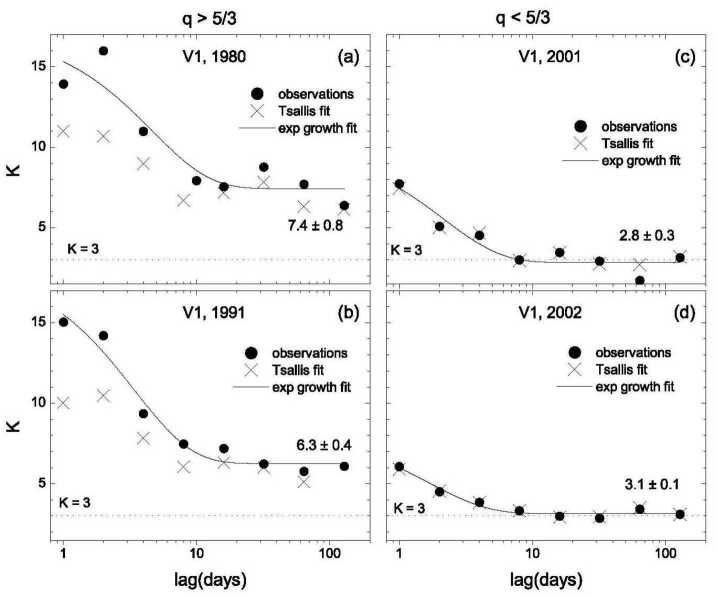
The kurtosis of the observations (solid circles) and of the Tsallis fits (x’s) closer to the sun (panels (**a**) and (**b**)) and farther from the sun (panels (**c**) and (**d**))

Figure 9 shows the kurtosis for the observations (solid circles) and the Tsallis fits (the exes, x) closer to the sun (panels (a) and (b)) and farther from the Sun (panels (c) and (d)). The solid curves are fits to the observations with an exponential growth curve. The values of the kurtosis were high during 1980 and 1991 when Voyager was within 45 AU of the Sun, decreasing from K = 15 to a plateau for scales greater than 10 days with a relatively large value, K = 7. During 2011 and 2012, when Voyager 1 was at 85 - 87 AU, the kurtosis was much smaller, decreasing from ∼K = 6 at a lag of one day to K ∼ 3 (a Gaussian distribution) for lags of 8 to 128 days.

This striking change in the behavior of the kurtosis at larger distances was also observed for the non-extensivity parameter, q. The Tsallis distribution had a *finite* variance when q < 5/3 and a *divergent* moment when q > 5/3. For the 1980 and 1991 data (near 8 AU and 45 AU, respectively), q > 5/3 at all scales from one day to 128 days, owing to large tails of the probability distribution functions (PDFs) caused by large fluctuations and jumps in B(t). In Tsallis statistical mechanics, the PDF describes the metastable or quasi-stationary state with the parameter q_stat_. By contrast, for the 2001 and 2002 data q < 5/3, and q approached 1 (the PDF approached a Gaussian) at scales greater than the solar rotation period. The values of q < 5/3 are a consequence of the smaller tails of the PDFs (smaller jumps in B) during 2001 and 2002. During 2001 and 2002, q tends to approach unity indicating that the distributions are becoming Gaussian. The transition from q > 5/3 at <45 AU to q < 5/3 at >80 AU suggests the possibility of a “phase transition” between 45 AU and 80 AU.

As shown in Fig. 8, the Voyager observations of B showed that the increments of the magnetic field strength by dBn(t_i_) = (B(t_i_) + t_n_) − B(t_i_))/〈B(t_i_)〉 on scales t$_{\mathrm{n}} = 2^{\mathrm{n}}$ days, where n = 0, 1, 2, 3, 4, 5, 6, and 7 and t_i_ is time can be described by the Tsallis distribution from 1 AU to 87 AU.

**Fig. 10:**
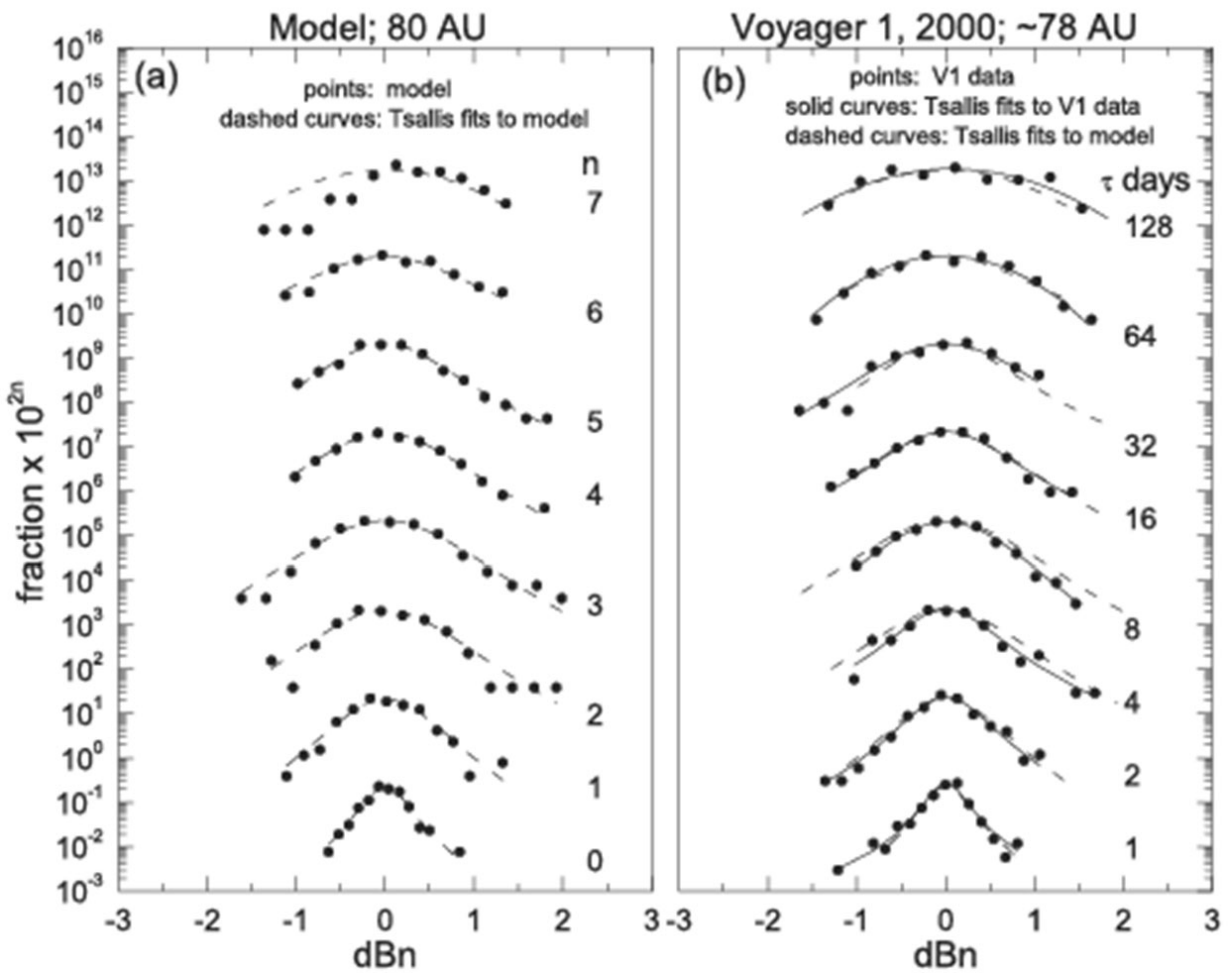
Model predictions and observations of the magnetic field distributions

A MHD model which includes pickup ions was used to calculate the radial evolution from 1 AU to 80 AU of the distribution of increments of the field strength $B$ on scales from 1 to 64 days (Burlaga et al. 2007). Plasma and magnetic field data from 1 AU were input into a 1-D MHD model. Figure 10 (left panel) shows the predictions of the model at 80 AU. The distributions of increments dBn of $B $ are shown by the dots and the fit to the observations with a Tsallis distribution is given by the dashed curves. The model distributions are well-described by Tsallis distributions on scales t$_{\mathrm{n}} = 2^{\mathrm{n}}$ days, where n = 0, 1, 2, 3, 4, 5, and 6, i.e. on scales from 1 to 64 days. The model correctly predicts that there is a significant deviation of the observations from the Tsallis distribution at the largest scale, 2^7^ days = 128 days. Thus, the 1-D MHD model predicts that the increments of B at 80 AU are described by the Tsallis distribution on scales from 1-64 days.

**Fig. 11:**
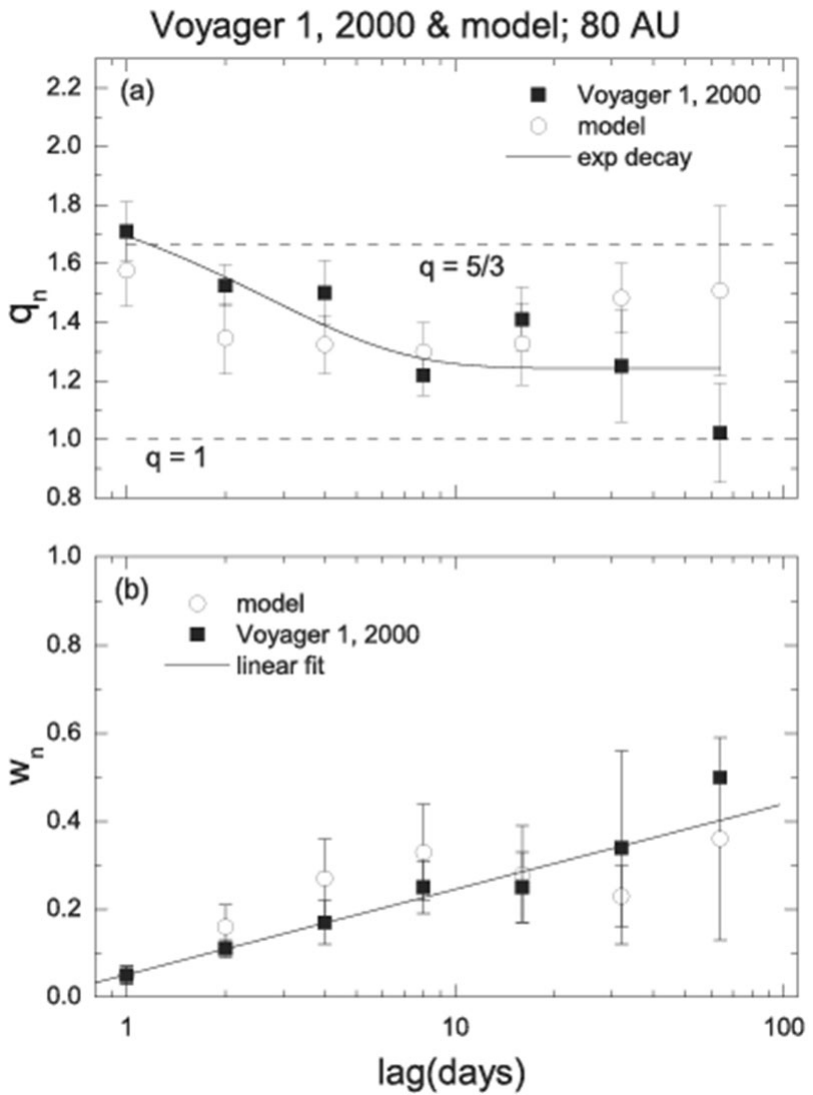
Top: entropic index q and width of the model and observed Tsallis distributions

The MHD model also predicts that the entropic index q decreases from q > 5/3 at R ∼ 45 AU to q < 5/3 at R ∼80 AU (Fig. 11a), indicating a change from a divergent to a convergent 2nd moment of the Tsallis distribution, as observed by Voyager 1. This result suggests the possibility of a “phase transition” or a relaxation effect from q > 5/3 at < 45 AU to q < 5/3 at > 80 AU. The model also predicts that the width w$_{\mathrm{n}} = 1$/(dBn)$^{1/2}$ of the Tsallis distribution increases linearly with increasing lag (Fig. 11b).

## The Termination Shock

The solar wind is super-magnetosonic in the inner heliosphere, so as it approaches the heliopause a termination shock forms where the plasma slows down, is compressed, heated, and becomes subsonic. Voyager 1 crossed the TS in 2004 at 94 AU and V2 crossed in 2007 at 84 AU; these crossings revealed the size of our heliosphere. TS effects were observed upstream of the crossings. Voyager 1 discovered the termination foreshock region starting at 85 AU, in which the energetic particle intensities increase when magnetic field lines are connected to the TS. The particles energized at the TS flow inward along these field lines and populate the foreshock. Voyager 2 entered the foreshock region at 75 AU, about 9 AU before the TS crossing. Electron plasma oscillations were observed weeks prior to the shock, as would be expected in an electron foreshock associated with the TS (see Fig. 14). The foreshock is a dynamic region since changes in the plasma pressure and magnetic field direction change the pathlength between the spacecraft and TS and thus the particle intensities.

**Fig. 12:**
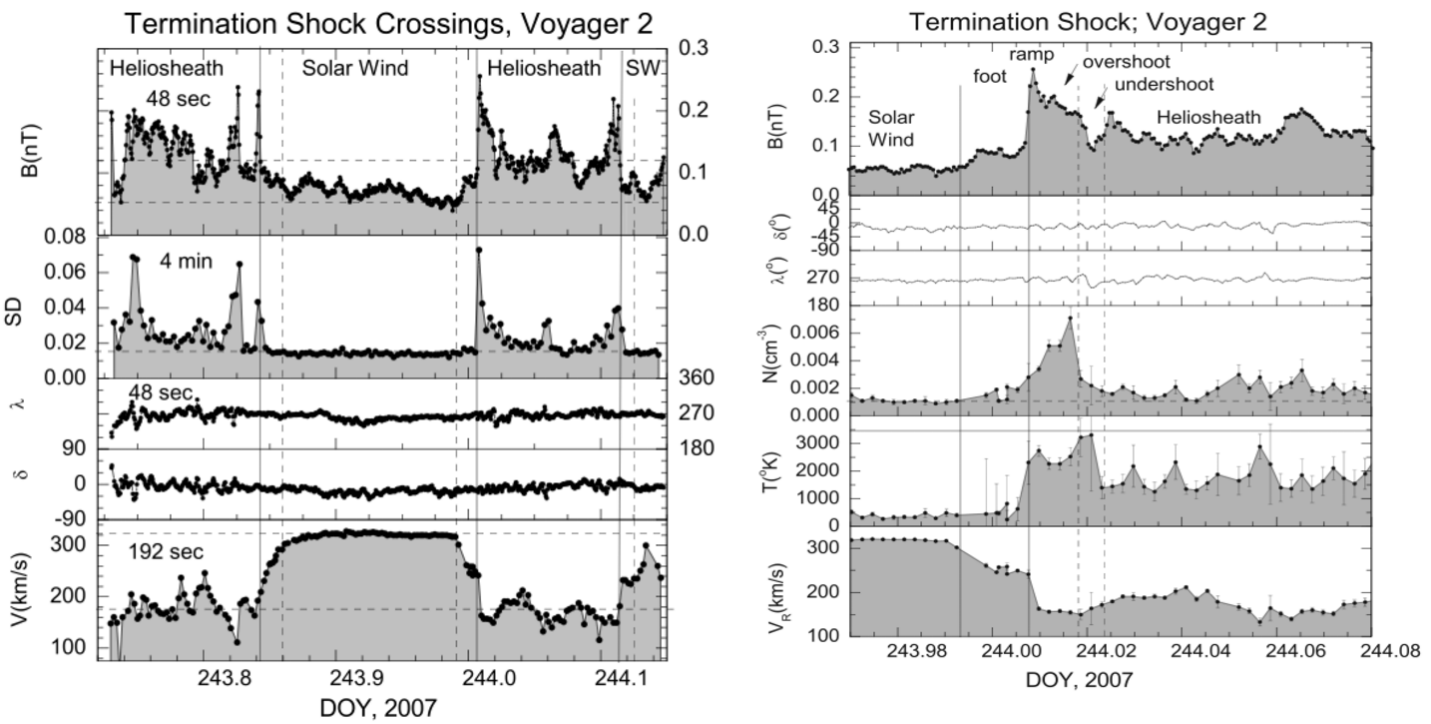
Plasma and magnetic field data at the TS (Burlaga et al. 2008; Richardson et al. 2008a). Left: the three TS crossings observed by V2 showing |B|, the standard deviation of |B|, the azimuthal and elevation angles of $\mathbf{B}$, and the plasma speed. Right: a blowup of the second TS crossing showing |B|, the azimuthal and elevation angles of $\mathbf{B}$, and the thermal proton density, temperature, and speed

V1 crossed the TS during a tracking gap; therefore, it did not observe the actual shock, but several observations indicated that V1 had entered the heliosheath. The magnetic field strength increased by a factor of ∼3, consistent with expectations for the TS, and the variability of $\mathbf{B}$ increased dramatically (Burlaga et al. 2005). The low-energy particle intensities jumped at the TS, but the higher energy anomalous cosmic rays (ACRs) did not (Decker et al. 2005; Stone et al. 2005). The first and last V2 TS crossing occurred in data gaps, but the TS moved across V2 three times while V2 had DSN tracking (Fig. 12), providing the first and only in situ TS data.

The first V2 termination shock precursor in the plasma data were the three step-like speed decreases, shown in Fig. 12, that started 90 days before the TS. V_R_ decreased from 400 to 300 km/s, removing ∼40% solar wind flow energy ahead of the shock. The last decrease, from 350 to 310 km/s, was coincident with a low-energy particle intensity increase; the inward pressure gradient from the particle increase is sufficient to produce the reduction in the solar wind speed (Florinski et al. 2009). Thus, the V2 TS is the first example of a particle-mediated shock, where the particles accelerated at the shock move upstream and change the shock structure.

The TS is dynamic and may reform on the scale of several hours. The TS looked like a classic super-critical, quasi-perpendicular shock in 2 of the 3 crossings (Burlaga et al. 2008); one is shown in Fig. 12. It has a foot region, formed by reflected ions, with larger B and lower V. The most rapid changes in the plasma and magnetic field were in the ramp region, which was followed by an overshoot of B and then fluctuations downstream. The high-resolution magnetic field data resolved the ramp structure, which had quasi-periodic fluctuations with length scales of about 1000 km (Burlaga et al. 2008). The other TS crossing was different (Fig. 12), with two ramp-like features that suggest the TS was reforming (Burlaga et al. 2008). The shock strength was 2-3 and the TS speed was 60-100 km/s, similar to the speeds of planetary bow shocks (Richardson et al. 2008a)

**Fig. 13:**
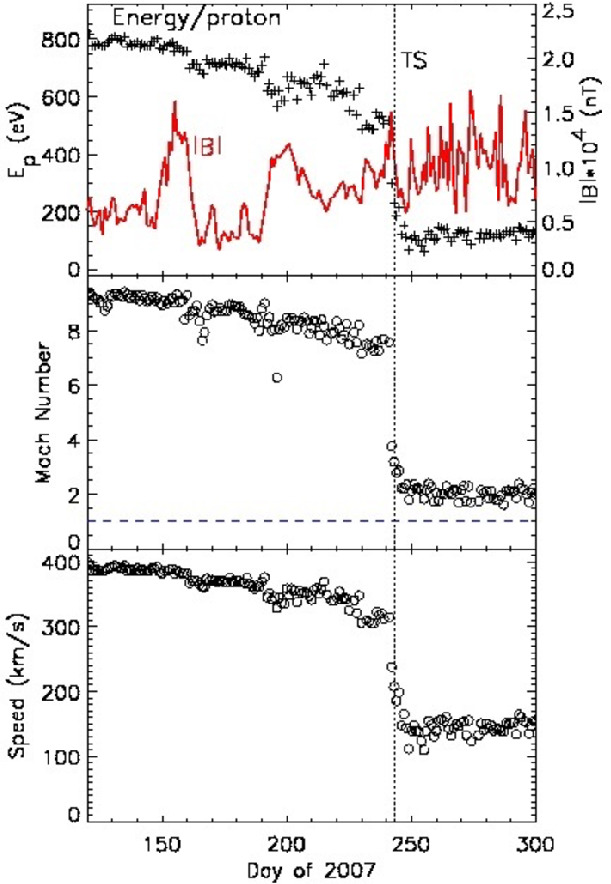
(**a**): the top panel shows the total (flow plus thermal) energy of the protons and |B| as V2 crossed the TS. The bottom panel shows the Mach number of the thermal protons remains > 1 after the TS. (**b**): the V2 speed profile across the TS (Richardson et al. 2008a)

A surprise from the V2 TS was that the thermal plasma downstream of the shock was only heated to $10^{5}~\text{K}$, compared with expectations of over $10^{6}$ K (Fig. 13). Only 20% of the solar wind flow energy heated the thermal solar wind plasma; most of the energy heated the PUIs (Richardson et al. 2008a). The solar wind thermal ions gain so little energy they remain supersonic in the heliosheath; the PUIs determine the sound speed, which is subsonic as required. At the TS, almost all the thermal solar wind ions pass directly through the shock potential (Zank et al. 1996), unlike at planetary bow shocks where up to 50% of the thermal ions are reflected (Richardson 1987). The PUI distribution is much broader in energy than the thermal ions and some of the PUIs are reflected. The flow energy of the solar wind goes mainly to the pickup ions. The PUI gain about 65% of the energy, thermal solar wind ions gain 20%, and more energetic ions gain 15% (Decker et al. 2008). However, energetic ions were accelerated only to lower energies. ACRs were not created at either the V1 or V2 TS crossing.

The TS is not symmetric in radial distance. The first evidences of asymmetry were the two crossings of the inner foreshock boundary. V1 entered the foreshock at 85 AU. When V1 crossed the TS at 94 AU, V2 was entering the foreshock region at 75 AU. The V2 TS crossing was at 84 Au, also 10 AU closer than the V1 crossing (Burlaga et al. 2008; Decker et al. 2008; Richardson et al. 2008a; Stone et al. 2008). Changes in the solar wind dynamic pressure account for only 2-3 AU of this difference. Models can reproduce the asymmetry if the VLISM magnetic field were of order 3-5 nT, much larger than the ∼1 nT pre-Voyager estimates but comparable to the observed VLISM B. The observed VLISM B near the HP was larger at V2 than V1, consistent with B being more compressed in the south due to the draping of the magnetic field. The field must also be at the right orientation to the LIC flow (Izmodenov et al. 2005; Opher et al. 2007; Pogorelov et al. 2009) to produce the asymmetry. As of 2021 (35 AU beyond the TS), the V1 magnetic field remains solar-like and the pristine field direction is not known. IBEX observations show the pressure is highest south of the HP nose, consistent with a closer TS and HP in the south (McComas and Schwadron 2014).

### Plasma Waves Associated with the Termination Shock

The Voyager PWS instruments observed plasma waves associated with the TS (Gurnett and Kurth 2005, 2008). Voyager 1 and 2 both observed electron plasma oscillations prior (upstream) to the TS crossing. At V1, these were observed up to ten months prior to the TS crossing whereas Voyager 2 observed them only about one month prior to the TS. The reason for this variation is not definitively known, but could be due to variations in the relative motion of the TS, the energy of the electron beams underlying the generation of the waves, and the specific geometry of the magnetic field connecting the TS to the spacecraft at the time the waves were observed. For both spacecraft, the plasma oscillations were detected in the 311 Hz spectrum analyzer channel that has a bandwidth of ±15%. The plasma oscillations occur at the electron plasma frequency $f_{pe} = 8980(n_{e})^{1/2}$, where frequencies in Hz and electron densities $n_{e}$ in cm^−3^. Hence, for 311 Hz, $n_{e}$ is $1.2 \times 10^{-3}$ cm^−3^ and is typical of plasma densities in the solar wind at 90 AU. An example of the Voyager 2 observations is given in Fig. 14 from Gurnett and Kurth (2008).

**Fig. 14:**
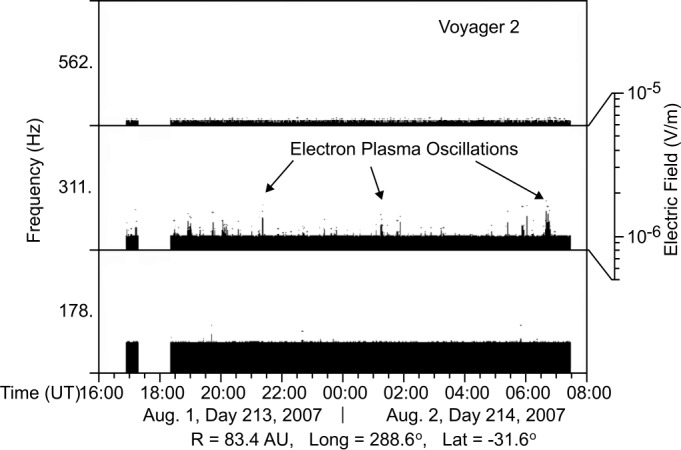
Electric field intensities in three of the V2 PWS spectrum analyzer channels on days 213 and 214 of 2007 obtained prior to V2 crossing the TS. The bursty emissions in the 311 Hz channel are electron plasma oscillations at the plasma frequency indicating an electron density of about $1.2 \times 10^{-3}$ cm^−3^ (from Gurnett and Kurth 2008)

**Fig. 15:**
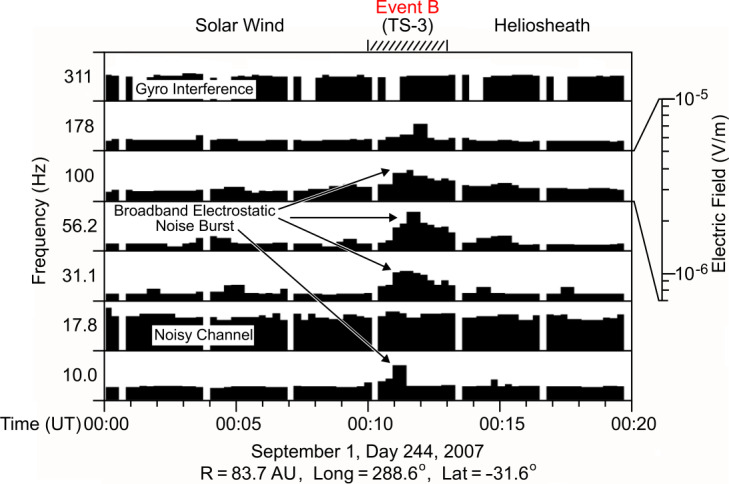
Electric field observations by Voyager 2 covering the frequency range from 10 to 311 Hz during a TS crossing on day 244 of 2007. The broadband signature starting at 00:11 corresponds to the ramp of the shock and is similar to the spectra observed at planetary bow shocks (from Gurnett and Kurth 2008)

While Voyager 1 crossed the TS during a data gap, Voyager 2 observed multiple crossings, allowing the detection of plasma waves associated with the shock. An example of the V2 TS plasma waves is shown in Fig. 15 (Gurnett and Kurth 2008). As in planetary bow shocks, the wave signature of the TS is a broad spectrum below the local electron plasma frequency. Voyager does not have a wave magnetic field sensor; however, by analogy with planetary bow shocks, it is likely this feature primarily comprises electrostatic waves. For two of the Voyager 2 TS crossings the broadband signatures corresponded well with the ramp of the shock, suggesting the currents in the ramp drove these waves.

## The Heliosheath

Investigating the global nature of the heliosheath, including its thickness and overall pressure balance, is fundamental to understanding the heliosphere as a whole. V1 and V2 found that the heliosheath pressure is dominated by suprathermal particles, which was verified by the Voyager Low Energy Charged Particle (LECP) and plasma subsystem (PLS) data (Decker et al. 2015), a combination of Voyager LECP particle data and Cassini/INCA energetic neutral atoms (ENAs) data (Dialynas et al. 2019), and recent MHD models (Opher et al. 2020). Rankin et al. (2019) calculate the total effective heliosheath pressure and compare it with IBEX observations to provide additional evidence that the heliosheath dynamics are driven by suprathermal energetic processes.

**Fig. 16:**
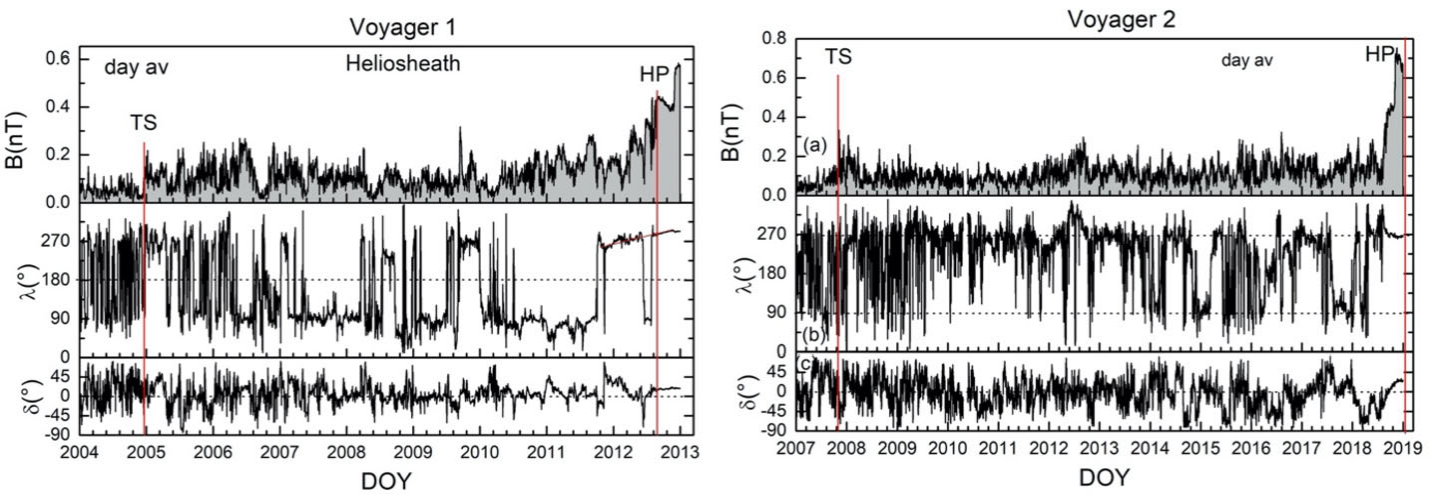
Daily averages of the V1 (left) and V2 (right) magnetic field magnitude, azimuthal angle, and elevation angle in the heliosheath

Voyager 1 moved through the heliosheath at northern heliolatitudes and Voyager 2 was in the south. Although they were both launched in 1977, they arrived at the termination shock at different times, 2004.95 and 2007.49, respectively. The magnetic field magnitudes and directions are shown in Fig. 16 (Burlaga et al. 2021a). The azimuthal angle, $\lambda $, of the magnetic field is scattered about 90° and 270° and the elevation angle, $\delta $, is scattered about 0°, as predicted by the Parker magnetic field model. A sector structure, with alternating inward and outward magnetic fields, was observed by both spacecraft, but Voyager 2 observed a long period of unipolar magnetic fields when it was below the heliosphere current sheet (HCS). The average B did not change until very close to the HP crossing at either V1 or V2. The magnetic field intensity was strongly influenced by solar activity. Voyager 1 observed more MIRs and GMIRs than Voyager 2. Relatively few weak MIRs were observed by Voyager 2 when it was at lower latitudes than the HCS. Relatively strong magnetic fields were observed at Voyager 1 and Voyager 2 for several months as the spacecraft approached the heliopause, when they moved through the magnetic barrier.

**Fig. 17:**
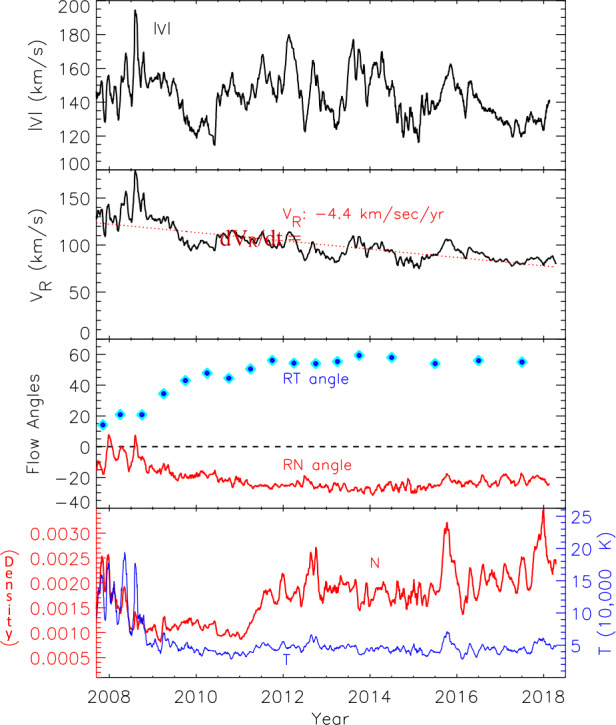
The V2 plasma speed, radial speed, flow angles in the RT and RN planes, density and temperature in the heliosheath

**Fig. 18:**
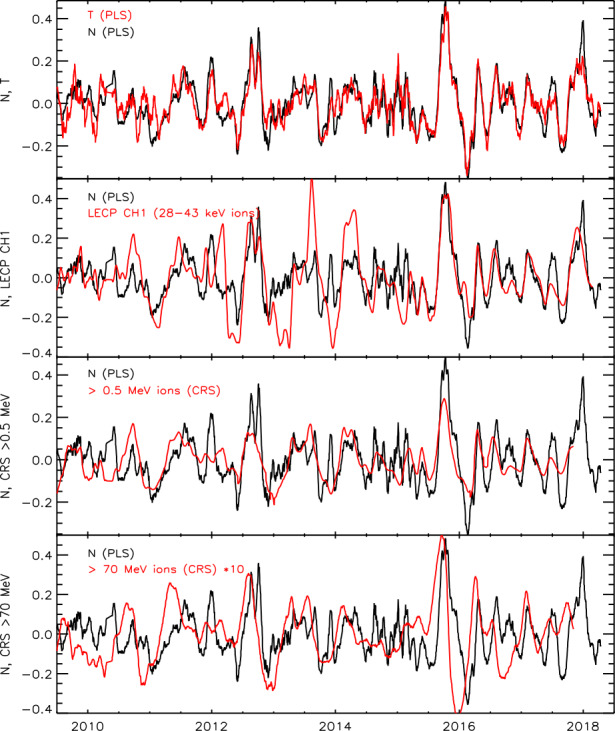
A comparison of the changes of plasma density, temperature, keV and MeV heliosheath ions, and GCRs. For each quantity x, $(\text{x}-\langle\text{x}\rangle)/\langle\text{x}\rangle$ is plotted where $\langle\text{x}\rangle$ is the running one-year average. GCR changes are multiplied by 10 but the other plots have no normalization

Figure 17 shows the V2 plasma velocity, density, and temperature across the heliosheath. The average speed |V| is roughly constant at 145 km/s until just before the HP, with excursions between 120 and 180 km/s. V_R_ decreases slowly from 130 km/s near the TS to 80 km/s before the HP boundary region, where it decreases more rapidly and then goes to zero at the HP. V_T_ increases away from the TS; it becomes hard to measure with PLS when the flow angle V surpasses 50° (beyond the field of view of the instrument) but the LECP data show it reaches 100 km/s near the HP. The RT angle increases to a plateau at about 55° in 2012 that extends to the HP. The flow angle out of the solar equatorial plane $\rho $ changes from −10° near the TS to −25° near the HP with a maximum flow angle of −30°. The density initially falls by a factor of 2 after the TS in 2009, then increases in 2011. This may be a solar cycle affect since the lower density region corresponds to solar minimum in the outer heliosphere. The average density then stays fairly constant from 2011 until it increases before the HP. The T drops from 150,000 °K near the TS to 50,000 °K by 90 AU and then stays, on average, constant to the HP. MIRs are observed at 99, 109, and 116 AU with increases in B, V_R_, N, and T. These are driven by solar transients and propagate through the heliosheath. After the 116 AU MIR, a series of pressure pulses are observed with increases in N, T and the energetic particle intensity. Figure 18 compares the changes in N, T, keV ions, MeV ions, and GCRs. The correlations are very good for the all the parameters, suggesting the heliosheath is traversed by pressure waves with alternating compression and rarefaction regions. Before the HP there is a rise in B, N, T, and a decrease in V_R_, as discussed in the HP section. These changes suggest that pressure waves move through the heliosheath compressing magnetic flux tubes and the plasma and energetic ions within by similar amounts.

### Flow Speeds in the Heliosheath

Since V1 does not have a working plasma instrument, the plasma density and temperature are not known and speeds are derived from particle anisotropies using the Compton-Getting (CG) effect (Compton and Getting 1935; Gleeson and Axford 1968; Forman 1970; Ipavich 1974). The CG method uses energetic particle fluxes from different look directions to derive plasma flow speeds. Krimigis et al. (2011) report that V1 entered a stagnation region in 2010, about 8 AU before the HP, where V_R_ went to 0 and was sometimes negative (inward) and V_T_ and V_N_ were small. The V1 CRS instrument observed > 0.5 MeV anisotropies during spacecraft rolls and derived V_R_ consistent with the LECP V_R_, also showing a stagnation region near the HP (Richardson et al. 2021). This stagnation region was unexpected; one possible explanation is that HP instabilities distort the flow field (Borovikov and Pogorelov 2014). An additional puzzle was that, despite the speed decrease, B did not increase and the magnetic flux was not conserved (Richardson et al. 2013, 2021).

**Fig. 19:**
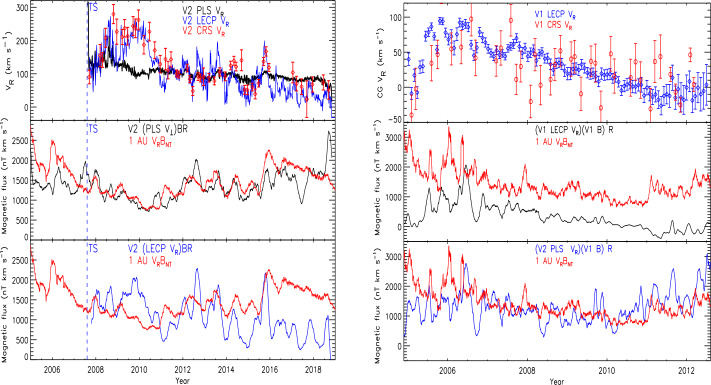
Left: the top panel shows the V2 radial speed measured by PLS (black) and derived from the CG effect using LECP (blue) and CRS (red) data. The middle and bottom panels compare the magnetic flux at 1 AU (Omni data set) with that calculated at V2 using the PLS and LECP CG speeds, respectively. Right: the top panel shows the V1 radial speed derived from the CG effect using LECP (blue) and cosmic ray subsystem (CRS) (red) data. The middle and bottom panels compare the magnetic flux at 1 AU (Omni data set) with that calculated at V1 using the PLS speeds and using the V2 LECP CG profile, respectively

The V2 PLS V_R_ profile was also surprising. V_R_ decreased only slowly until the HP crossing. The LECP V2 CG V_R_ profile derived from 28-53 keV ion anisotropies differs from the PLS V_R_ (see Fig. 19); PLS shows a slow steady decrease while LECP shows large, >100 km/s, excursions in V_R_. Starting in 2016, about 8 AU before the HP, the LECP CG V_R_ averaged about half that observed by PLS. Krimigis et al. (2019) suggest this slowdown region is analogous to the V1 stagnation region. The V2 CRS V_R_ using >0.5 MeV ions are again consistent with the LECP V_R_ (Cummings et al. 2021). These authors suggest that the CG calculations are flawed since they disagree with the direct plasma observations; however, another possibility is that PLS speeds are not correct. Richardson et al. (2021) show that if the PLS V_R_ profiles are used to calculate the magnetic flux, a conserved quantity (Parker 1963), then the magnetic flux at 1 AU and at V1 and V2 are similar. However, if the CG V_R_ profiles are used then the magnetic flux in the heliosheath differs greatly from that at 1 AU. They also show that if the V1 V_R_ profile were the same as that at V2, then magnetic flux conservation would hold at V1 as well. It is not understood why the different energy particle anisotropies from LECP and CRS give the same incorrect V_R_ values, but the V2 speeds and magnetic flux conservation argue against the presence of a stagnation region.

### Reconnection in the Heliosheath

**Fig. 20:**
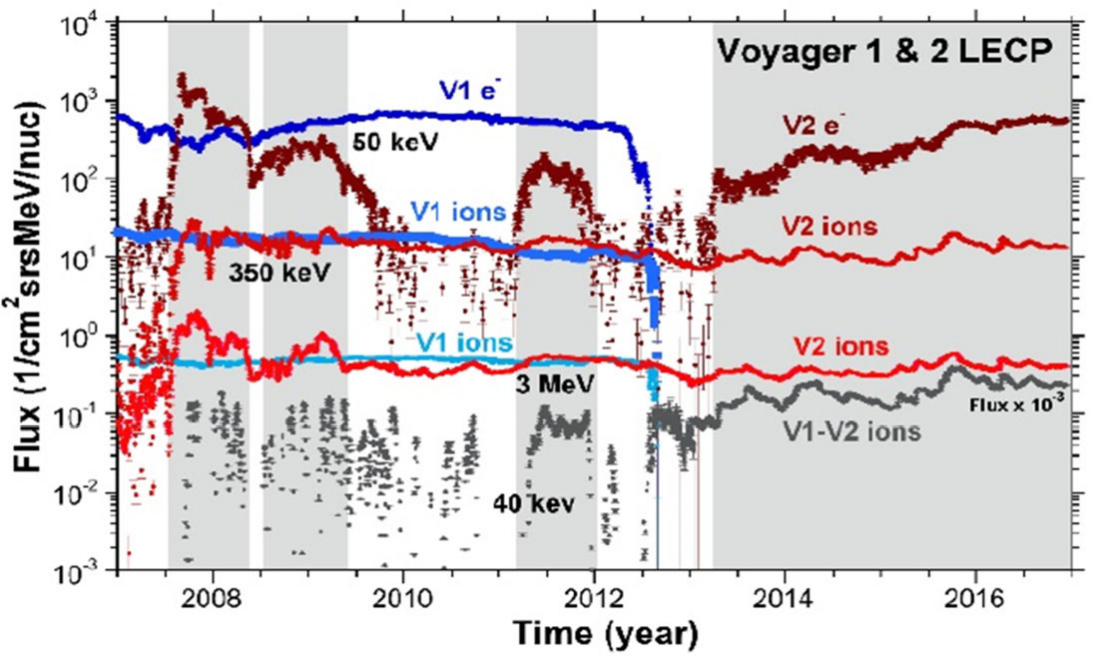
V1 and V2 LECP observations in the heliosheath for different energy ions and electrons. The white regions are where V2 was in a unipolar magnetic field region (below the heliospheric current sheet) and the gray regions are where V2 was in the sector region and saw both toward and away magnetic field directions. V1 is always in the sector region (Hill et al. 2014)

The compression of the heliosheath as it approaches the HP brings oppositely directed magnetic field lines together and could drive reconnection in the heliosheath (Lazarian and Opher 2009; Drake et al. 2010; Pogorelov et al. 2009, 2013), but the importance of this process is not clear. Opher et al. (2011) suggest that near the HP the heliosheath is comprised of magnetic bubbles formed by reconnection. Observations of the particle intensities in Fig. 20 show that in the unipolar (no reconnection) zone V2 LECP electron fluxes drop out and ion fluxes decrease (Hill et al. 2014). These fluxes are high in the sector zone where oppositely directed magnetic fields may reconnect and create magnetic bubbles that trap particles and keep the intensities high. Drake et al. (2017) predict B and N are correlated in the sector zone, but not in the unipolar zone, and data substantiate this prediction. However, the agreement of the magnetic flux values at 1 AU and in the heliosheath and the paucity of observations of magnetic D-sheets (Burlaga and Ness 2010; Burlaga et al. 2021b) argue against significant reconnection. The role of reconnection in the heliosheath is still not well understood.

### Transients Reaching Beyond the Heliosphere

The Sun is very active and drives transient structures with effects that persist through the heliosphere and into the LISM. The largest of these events are coronal mass ejections (CMEs) which propagate through the heliosphere as interplanetary CMEs (ICMEs). ICMEs expand outward to about 15 AU until they reach equilibrium with the surrounding solar wind (von Steiger and Richardson 2006). ICMEs have been tracked through the heliosphere to the TS. These ICMEs can drive shocks which persist out to the HP (Kim et al. 2017; Liu et al. 2014; Gurnett et al. 2013). Corotating interaction regions (CIRs) form when high speed solar wind streams overtake slower streams, with a forward-reverse shock pair forming at the interface. Both ICMEs and CIRs accelerate particles; ICMEs are more likely to drive large events, MIRs and GMIRS, that influence the outer heliosphere. More ICMEs occur at solar maximum. These ICMEs merge, with faster ICMEs overtaking slower ones, to form MIRs. These MIRs are quasi-periodic in the outer heliosphere near solar maximum, occurring roughly twice a year, with pressure enhancements of factors of 5-10. These MIRs run into the TS and move it outward, sending pressure pulses through the heliosheath. Figure 17 shows that these pressure pulses are characterized by highly correlated increases in the plasma density, pressure, and keV to MeV particle intensities as the whole region is compressed. The exception is B, whose magnitude is only weakly correlated with the pressure changes. These heliosheath pressure pulses eventually reach the HP, push the HP outward, and drive shocks into the LISM. These shocks generate electron beams which drive the plasma oscillations observed by PWS.

### Thickness of the Heliosheath

The thickness of the heliosheath from the TS to the HP was 28 AU at V1 and 36 AU at V2. Steady state MHD simulations give widths of 55–65 AU (see Kleimann et al. 2022, this journal). This difference implies that time dependence is important or that pressure is missing from the heliosheath; mechanisms suggested to resolve this problem are solar cycle variations (Izmodenov et al. 2005, 2008), removal of hot heliosheath ions by charge exchange (Malama et al. 2006; Opher et al. 2020), inclusion of thermal conductivity (Izmodenov et al. 2014), and escape of ACRs across the HP (Guo et al. 2018). All produce insufficient heliosheath thinning, as discussed in Kleimann et al. (2022, this journal)

### Heliosheath Magnetic Fields and Plasma: Solar Maximum

To contrast the magnetic observations in the heliosheath with those in the supersonic solar wind we discuss V2 observations from 2015, when V2 moved from 106.81 AU to 109.93 AU, latitude 30.9° S to 31.3° S and longitude 217.7° to 217.9° (Burlaga and Ness 2013). The average B during 2015 was 0.126 nT, which is higher than the heliosheath average, because in 2015 Voyager 2 was observing solar maximum. At least 3 MIRs were observed during the first 140 days of the year and a GMIR was observed from day 260 to at least day 305. It is noteworthy that such strong interaction regions, with B exceeding 0.2 nT, survive out to distances approaching that of the heliopause.

**Fig. 21:**
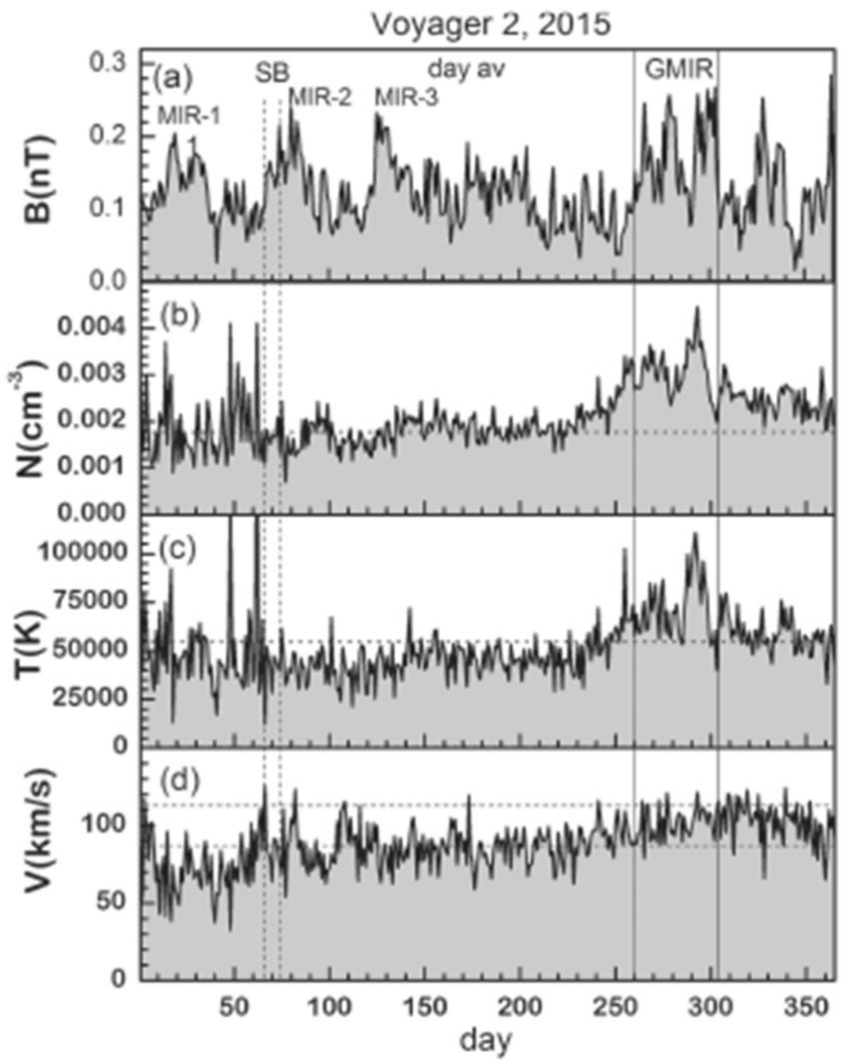
V2 magnetic field and plasma observations in the heliosheath in 2015

In the GMIR in Fig. 21, the density and temperature were enhanced significantly and the speed increased, suggesting that the GMIR was still growing in strength. The GMIR was related to a large increase in the dynamic pressure, NV^2^, that might have produced a shock in the VLISM. The GMIR produced a large decrease in >70 MeV/nuc GCRs, as typically observed through the heliosheath and heliosphere beyond 10 AU (Burlaga et al. 1985).

**Fig. 22:**
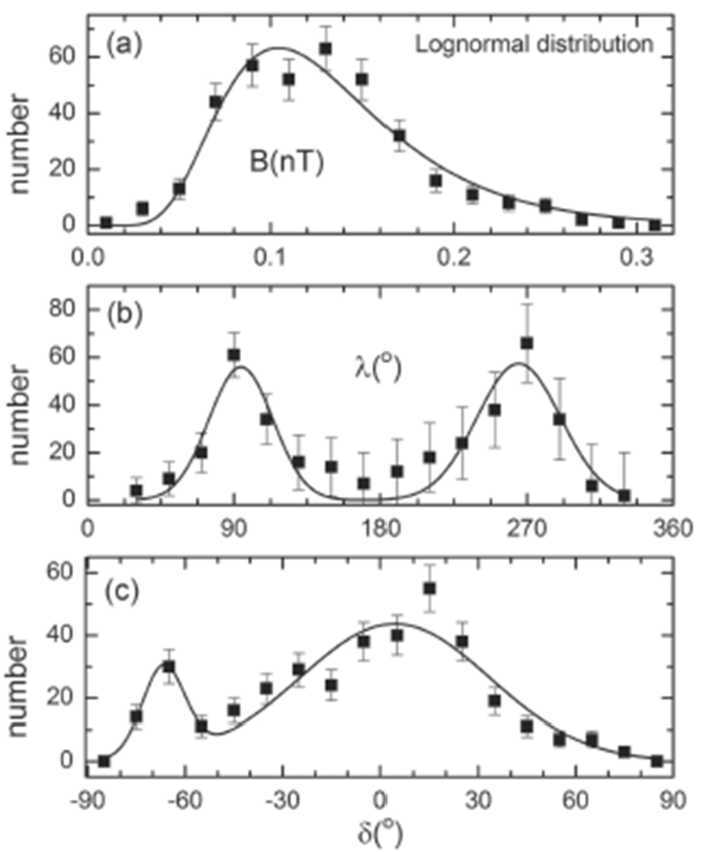
The distribution of V2 heliosheath magnetic field strengths and directions in 2015

Figure 22 shows that the distribution of the magnetic field strength during 2015 was lognormal, which is characteristic of the distribution of B that is observed when the sun is active and produces ejecta and magnetic clouds that interact with one another to produce the strong magnetic fields in MIRs and GMIRs. The distribution of the azimuthal angle $\lambda $ has two nearly equal height peaks at $\lambda = 90$° and 270°, corresponding to the Parker spiral magnetic field sector directions. The two peaks of $\lambda $ indicate that the heliospheric current sheet extended to large latitudes in the southern hemisphere, relative to the latitude of Voyager 2, and was warped, producing four sectors in the magnetic field. The well-defined sector boundary that V2 crossed near day 70, 2015 was thick; the change in the direction of $\boldsymbol{B}$ lasted ∼7 days. A minimum variance analysis showed that $\boldsymbol{B}$ rotated smoothly through a current sheet in a plane that was tilted 47° with respect to the solar equatorial plane. The velocity did not change when Voyager 2 crossed the current sheet, consistent with a tangential discontinuity. The distribution of the elevation angle, $\delta $, peaked at $\delta \sim 0$°, as expected for a spiral magnetic field. There was a change in $\delta $ to near −65° during an extended interval between days 150 and 210. These unusual angles might be attributed to a ripple in the heliospheric current sheet, or could be artifacts due to uncertainties in the calibration of the magnetic field.

**Fig. 23:**
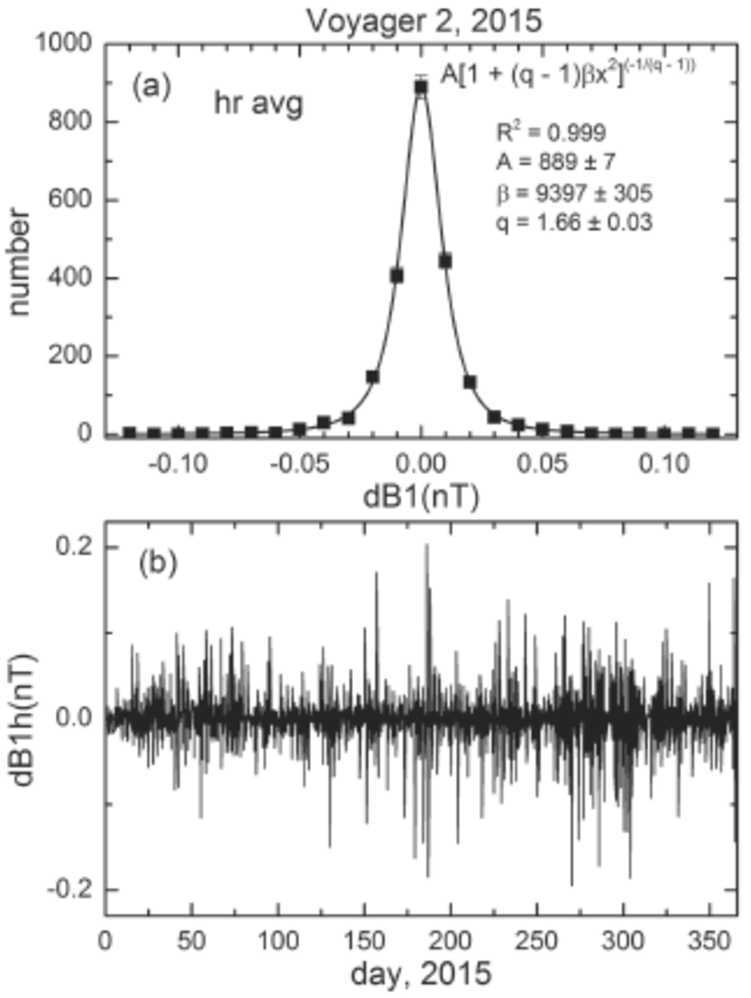
Increments of B: (**a**) distribution (dots) and fit to a Tsallis distribution (line) (**b**) time series

The small-scale structure of the V2 2015 magnetic field can be quantified by plotting the increments of B(t) on a scale of one hour, dB1h (t) = B (t + 1 hr) − B(t). Figure 23(b) shows that dB1h(t) for the one-hour of B was very bursty. The dots in Fig. 22(a) show the distribution of the increments of dB1h(t). The Tsallis distribution provides an excellent fit to the distribution of increments (the coefficient of determination R$^{2} = 0.999$), with a non-extensivity parameter q = 1.66 ± 0.03. Similar results for the distribution of daily averages give a fit to the Tsallis distribution with R$^{2} = 0.979$ and q = 1.60 ± 0.17. Thus, on these small scales, the non-extensively parameter during the undisturbed conditions shown in Fig. 23 is similar to that observed during the quiet solar wind conditions in 2000 discussed earlier.

### Heliosheath Magnetic Fields and Plasma: Solar Minimum

The non-extensive statistical mechanics of (Tsallis 1988, 2004a, 2004b, 2009) provide a description of the driven, open, non-equilibrium systems in the supersonic solar wind and heliosheath. This section discusses V2 observations of the 2010 “quiet” heliosheath magnetic field, when the spacecraft moved between 91.02 and 94.5 AU at latitudes 28.8° S to 29.3° S (Burlaga and Ness 2013). The magnetic fields were carried by solar wind that left the southern coronal hole on the sun during 2008 and 2009, when solar activity was historically low (Ahuwalia and Ygbuhay 2011) and B at 1 AU was only 4.0 nT. Voyager 2 was south of the heliospheric current sheet (which was relatively close to the solar equatorial plane, since it was solar minimum) and it measured a relatively weak average magnetic field of 0.08 ± 0.04 nT. Thus, Voyager 2 observed a “minimum energy state” of the heliosheath during 2010.

**Fig. 24:**
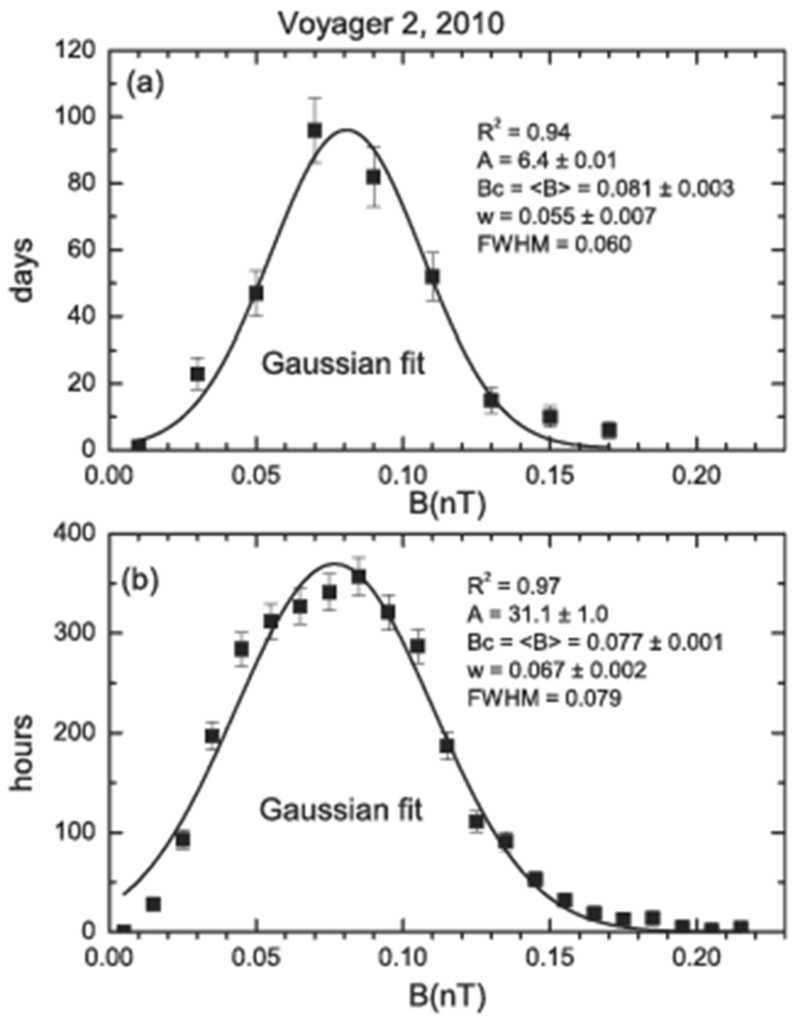
Distributions of daily (top) and hourly averages (bottom) of B. The squares show the data with 1$\sigma $ error bars and the lines show the Gaussian fits

The distributions of daily and hourly averages of B are shown in Figs. 24. The maximum of B was 0.08 nT for both distributions, very close to the average magnetic field strength. The full width at half maximum was 0.060 nT for daily averages and 0.079 nT for the hourly averages. Both B distributions in Fig. 24 are accurately described by a Gaussian distribution (with coefficients of determination R$^{2} = 0.94$ and 0.97, respectively), which is characteristic of B in the solar wind and the heliosheath when solar activity is very low.

Tsallis (2004a,b) introduced the concept of the “q-triplet” = (q_stat_, q_sen_, q_rel_) associated with the “non-extensively index” q_stat_ obtained from the Tsallis distribution of increments of B, the parameter q_rel_ related to the correlation coefficient, and the parameter q_stat_ derived from the multifractal spectrum. Burlaga and Vinas (2005a) were the first to compute the q-triplet for a physical system, using solar wind data. They obtained the values q_stat_ = 1.75 ± 0.06, q_sen_ = −0.6 ± 0.02, and q_rel_ = 3.8 ± 0.034 for observations made in the solar wind near 30 AU in 1989 and near 85 AU in 2002. They showed that the q-triplet had the properties associated with non-extensive statistical mechanics. For example, the deviation of the 3 q’s from unity is a measure of the departure from thermodynamic dynamic equilibrium. Nevertheless, the fluctuations in the solar wind and heliosheath are in a metastable, quasi-stationary state.

**Fig. 25:**
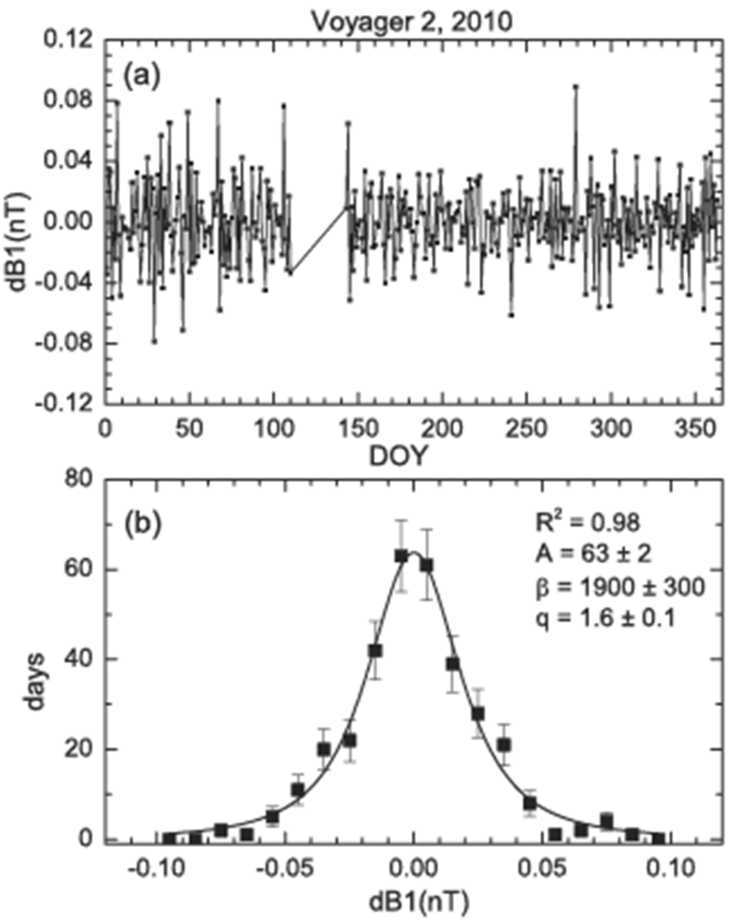
One-day increments of B, where dB1d = B(t + 1day) − B(t). Top: time series. Bottom: observed distribution (squares) and fit (line) using a Tsallis distribution

The one-day increments of B, dB1d = B(t + 1 day) − B(t), plotted in Fig. 25, show that the increments are “spiky” or intermittent. An excellent fit (R$^{2} = 0.98$) to the observations (the squares in Fig. 25b) was obtained with the Tsallis (q-Gaussian) distribution, shown by the solid curve, and the value q = 1.6 ± 0.01 was obtained from the fit. This value of q is the same as that observed during solar maximum conditions during 2015 discussed in Sect. 2, even though in 2010 the sun was inactive, with few sunspots!

As a measure of the significance of q = 1.6, we note that q = 1 for a Gaussian distribution and q = 1.7 is associated with the onset of chaos in the z = 2 logistic map (Tsallis 2009, p. 1964). The non-extensively parameter q is seldom much larger than 1.6 in the heliosheath and solar wind. The parameter q derived from the distribution of increments of B is the same as the parameter q_stat_. In Boltzmann Gibbs statistical mechanics, the PDF describes the thermal equilibrium state characterized by the temperature T.

**Fig. 26:**
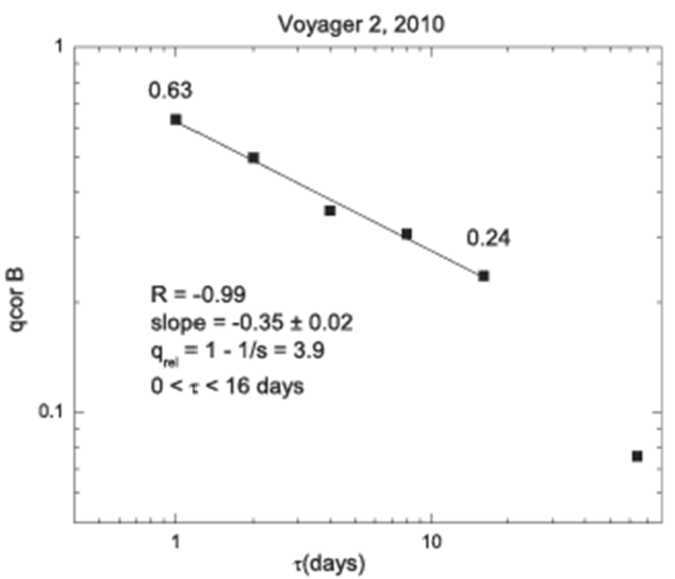
The correlation function for different time increments

In Boltzmann Gibbs statistical mechanics there is an exponential relaxation time to thermal equilibrium (exponential decay, with a relaxation time $\tau $). Tsallis statistics are associated with the q-exponential relaxation of microscopic quantities toward thermal equilibrium (q-exponential decay), with a relaxation parameter q_rel_ that can be computed from the “correlation function” C($\tau $) = q_rel_ $=\ \langle(\text{B}(\text{ti} + \tau ) - \langle \text{B(ti)}\rangle) \times ((\text{B} (\text{ti} + \tau ) - \langle \text{B}(\text{ti})\rangle) \rangle/\langle(\text{B}(\text{ti}) - \langle \text{B}(\text{ti})\rangle)2\rangle$ that is plotted as a function of $\tau $ on a log-log scale in Fig. 25. The result is accurately described (R$^{2} = -0.99$) by a linear fit with a slope s = $-0.35 \pm 0.02$ on scales from 1 to 16 days. Thus, we find that q_rel_ = 1 − 1/s = 3.9 (Tsallis 2009, p. 156). This result is in good agreement with the results that q_rel_ = 3.98 in 1989 and q_rel_ = 3.54 in 2002 obtained by Burlaga and Vinas (2005b). In Boltzmann-Gibbs statistical mechanics, the correlation function is an exponential, rather than a power law, related to q_cor_ in Fig. 26 as predicted by non-extensive statistical mechanics which we find in the Voyager 2 data.

**Fig. 27:**
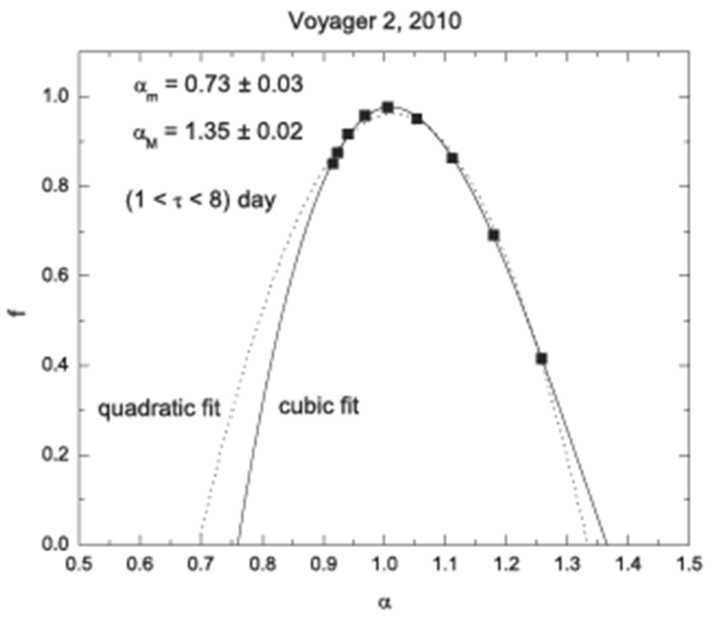
The multifractal spectrum of daily averages of B

In Boltzmann Gibbs statistical mechanics there is exponential sensitivity to the initial conditions (strong chaos, described by an exponential growth characterized by zero Lyapunov exponents and a growth parameter q_sen_). In Tsallis statistics the system is related to q-exponential sensitivity to the initial conditions described by q-exponential growth. This parameter is derived from the “multifractal spectrum” (Burlaga et al. 1993, 2006; Halsey et al. 1986; Hentschel and Procaccia 1983; Macek 2007; Macek and Szczepaniak 2008; Nauenberg 2003; Pirraglia 1993; Stanley and Meakin 1988; Tel 1988; Tsallis 2004b, 2009, and Tsallis and Brigatti 2004). The multifractal spectrum of daily averages of B observed by Voyager 2 during 2010 was computed using the methods described by Burlaga (1995) and Burlaga et al. (2006), and the results are plotted as points in Fig. 27 for $1 < \tau < 8$ days, where $f $ is the multifractal distribution function, which is a function of the parameter $\alpha $. The relevant quantities are the values of $\alpha $ at f = 0, but the observational points do not extend to f = 0.

The observations were extrapolated to f = 0 by means of the quadratic and a cubic fit. Using the cubic fit one obtains the minimum $\alpha \mbox{m} = 0.73 \pm 0.03$ and the maximum $\alpha \mbox{M} = 1.35 \pm 0.02$, similar to the values obtained by Burlaga and Ness (2012) in the heliosheath. From these numbers one obtains q_sen_ from the relation 1/(1 − q_sen_) = 1/$\alpha \min - 1$/$\alpha \max$ (Lyra and Tsallis 1998), hence $\text{q}_{\text{sen}} = -0.5 \pm 0.3$, compared with the average value for the distant heliosphere $\text{q}_{\text{sen}} = -0.6 \pm 0.2$ obtained by Burlaga and Vinas (2005a).

In summary, during 2010 Voyager 2 observed a 1) q-Gaussian distribution of daily increments of B with q = 1.6, 2) a power law correlation (rather than an exponential correlation) on scales from 1 to 16 days with q_rel_ = 3.9, and 3) a multifractal structure of B on scales from 1 to 8 days with q_sen_ = $-0.5 \pm 0.3$.)

**Fig. 28:**
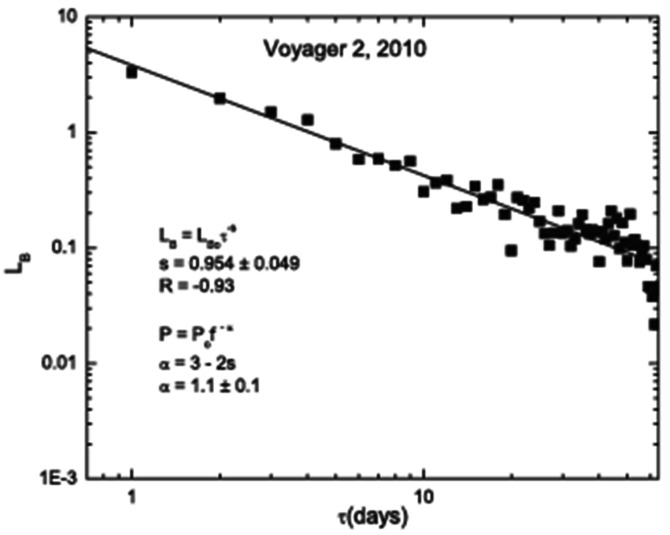
The length of the curve (B(t), $\tau $) as a function of lag $\tau $

Figure 28 (Burlaga and Ness 2013) shows that the length of the curve (B(t), $\tau $) (which is treated as a fractal) as a function of lag $\tau $ in a log-log plot is a straight line ($\text{R} = \pm 0.93$) on scales from $\tau = 1$ to 100 days. Thus, the length of the curve is a power law $\text{L} \sim \tau $^−s^, which corresponds to a frequency spectrum $\sim \text{f}^{-\alpha }$ where $\alpha = 3 - 2\text{s} = 1.1 \pm 0.10$ consistent with a “1/f” spectrum on scales from 1 to 100 days. This spectrum corresponds to an equal distribution of energy at all scales. This 2010 observation is the first demonstration of an f^−1^ spectrum associated with B in the heliosheath (Burlaga and Ness 2013).

## The Heliopause Region

**Fig. 29:**
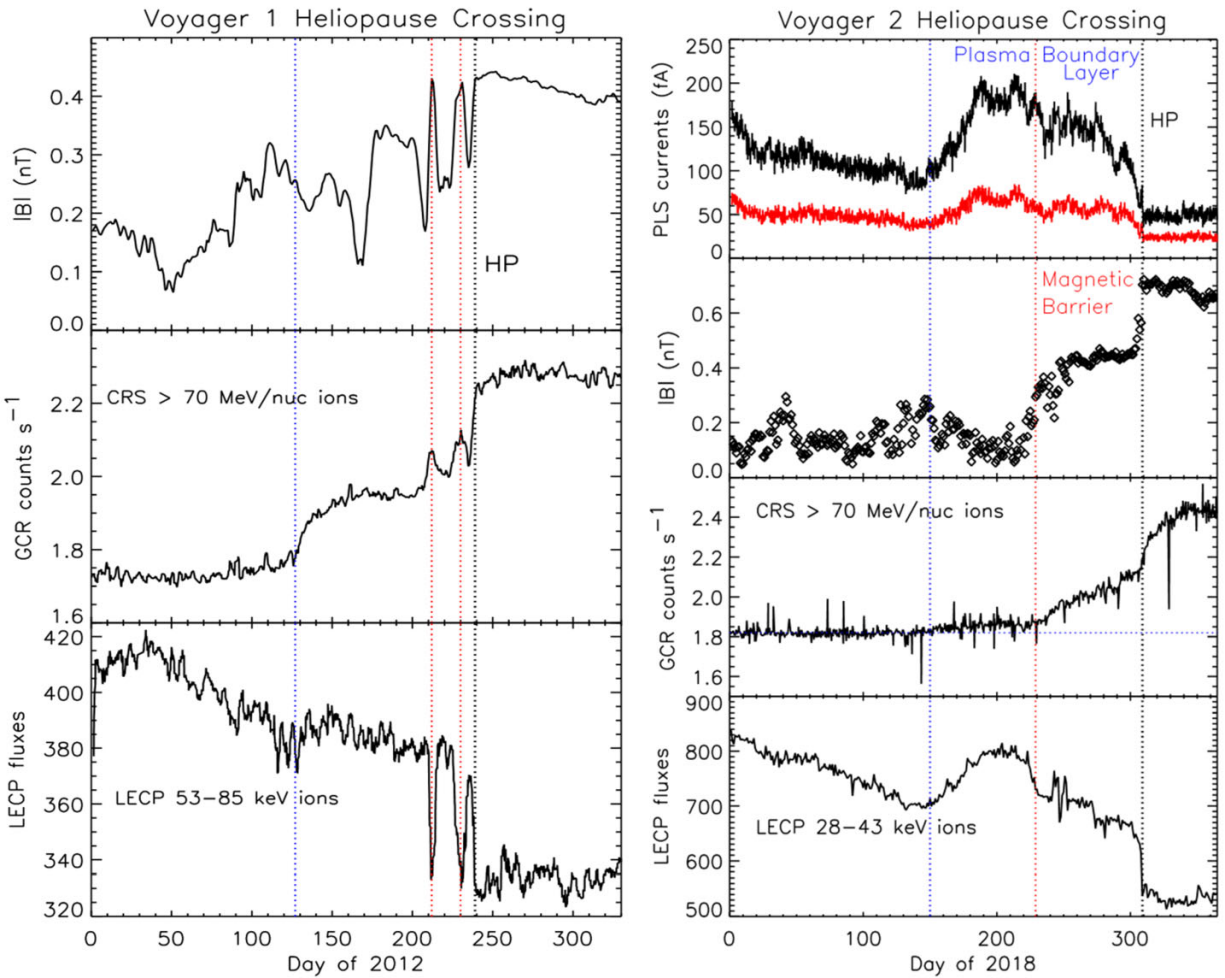
Overviews of the V1 (left) and V2 (right) HP crossings. The left panel shows B, the GCR intensity, and the LECP 53-85 eV ions. The right panel is similar, but the top panel shows that the currents measured by the B and C detectors on the plasma instrument drop out at the HP

The HP is the boundary between the solar wind plasma and the interstellar plasma. The location and nature of the HP were unknown before the Voyager crossings; it was thought to be a sharp tangential discontinuity in analogy with planetary bow shocks. This section describes the HP and its precursors upstream in the heliosheath. Figure 29 shows overviews of the HP regions at V1 and V2. V1 crossed the HP on day 238 of 2012 at 121.7 AU. V2 crossed it on day 318 of 2018 at 119.0 AU. The first clear evidences of the V1 HP crossing were an abrupt increase in magnetic field strength $B$, a decrease of heliosheath energetic particles, and an increase in the GCR counting rate at 2012.56 (Stone et al. 2013; Krimigis et al. 2013; Burlaga et al. 2013). These changes were followed by a recovery, a larger abrupt change, another recovery, then a final crossing after which $\boldsymbol{B}$ remained high and steady, the energetic particles disappeared, and the GCR intensities plateaued (see region T near 2012.6 in Fig. 29). Figure 31 shows that the direction of $\boldsymbol{B}$ did not change across the HP, contrary to expectations that the VLISM field would be tilted with respect to the solar field. The steady V1 $\boldsymbol{B}$ direction caused controversy about whether this boundary was the HP or a transition from closed field lines to field lines connected to the LISM, allowing heliosphere ions to escape into the LISM and GCRs to enter the heliosphere. The strong uniform magnetic fields, absence of energetic particles, the plateau of the GCRs, and the subsequent observation that the density was 0.08 cm^−3^ (Gurnett et al. 2013) were strong evidence that V1 had crossed the HP, but other hypotheses persist (Gloeckler and Fisk 2015).

The V2 HP crossing had similar features, an increase in GCR intensity, an increase in B, and a decrease in heliosphere ion intensity (Stone et al. 2019; Krimigis et al. 2019; Burlaga et al. 2019). The magnetic field direction again did not change. V2 has a working plasma instrument which observed a sharp change in the plasma flux at the HP (Richardson et al. 2019). The higher densities in the VLISM were again confirmed by PWS (Gurnett et al. 2021).

**Fig. 30:**
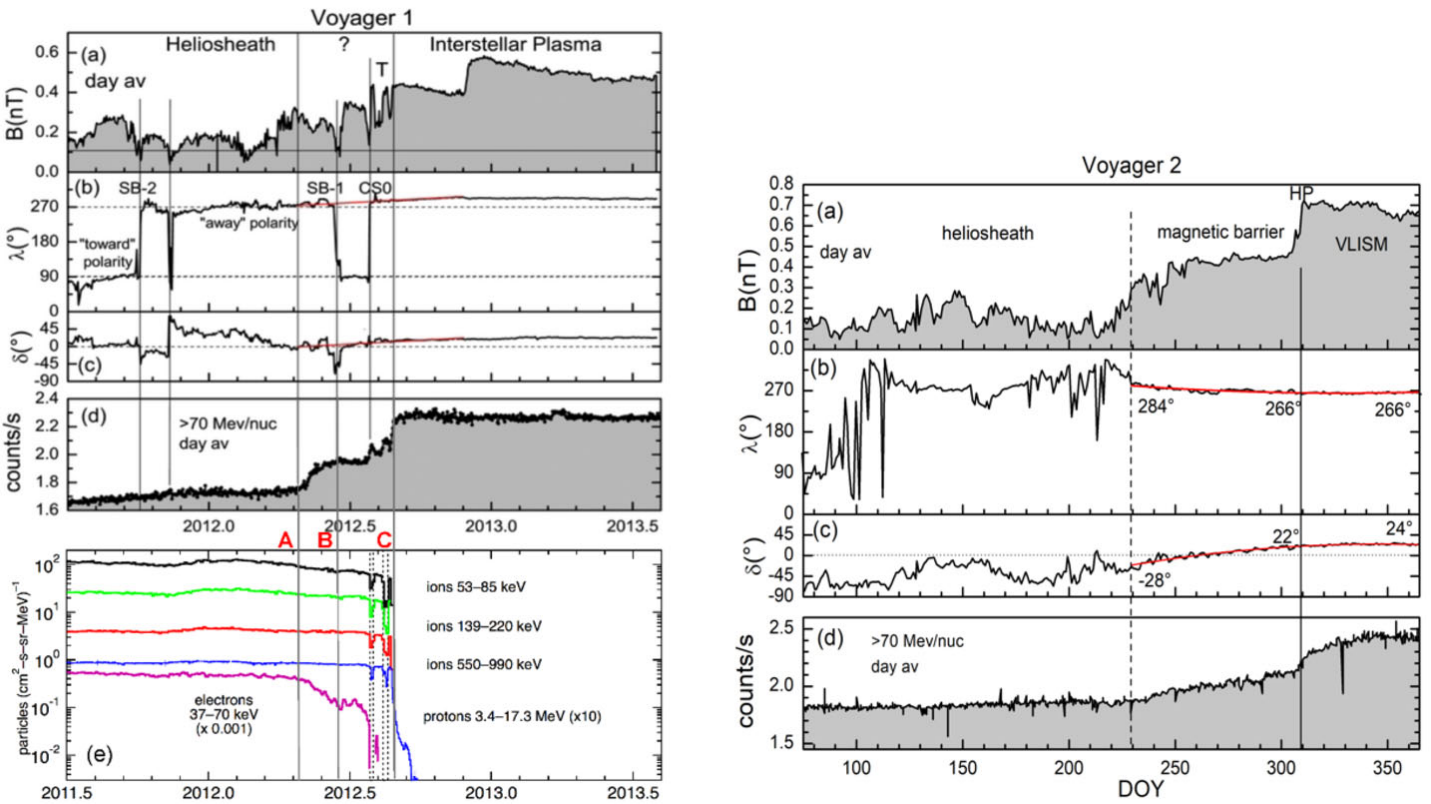
The magnetic field observations at the HP for V1 (left) and V2 (right). The panels are B, the azimuthal and elevation angles of $\boldsymbol{B}$, and the GCR intensities. The left bottom panel shows LECP energetic particle intensities

V1 and V2 observed HP boundary regions and complex structure before the HP crossings. Figure 30 compares the V1 and V2 HP crossings to illustrate which features are common and which may depend on time and/or position. The first precursor of the HP may be the decrease in CG V_R_ speed at both V1 and V2 shown in Fig. 18 about 8 AU before the HP (Krimigis et al. 2013, 2019). As discussed above, neither B nor the PLS V_R_ at V2 change at this boundary; the cause of the CG speed decrease is not known but may be a HP boundary effect.

### The Heliopause Boundary Region and Magnetic Barrier

The V1 HP crossing is preceded by the HP boundary region, a ∼1.3 AU wide region that started at day 125 of 2012 with a step increase in the GCR rates and a decrease in heliosheath electrons (Fig. 29) and ended at the HP with a second step increase of GCRs, an increase in B, and a dropout of low-energy ions to near background levels (Webber and Mcdonald 2013; Krimigis et al. 2013; Stone et al. 2013; Burlaga et al. 2013). The magnetic field lines in this region may be reconnected to the VLISM magnetic field enabling GCRs to enter the heliosheath and heliosheath electrons to exit.

V2 observed a similar boundary region with an increase in the GCR intensity and decrease in heliosheath electron flux on day 229 of 2018, so open field line boundary regions may be a common feature at the HP. At V2, the GCR increase was coincident with the beginning of the magnetic barrier, a region of increased B not observed at V1. The average B in the V2 magnetic barrier was $\sim 0.40 \pm 0.06$ nT, significantly greater than the average B of ∼0.13 nT from days 1–229 of 2018. The magnetic barrier had the strongest magnetic fields observed in the heliosheath and persisted for 80 days, from day 229 to day 309; these fields were comparable in strength to the magnetic field in the VLISM observed by Voyager 1. Neither the plasma parameters nor the LECP ion intensity changed at the start of the magnetic barrier. The V2 magnetic field increased to 0.7 nT at the HP, significantly larger than the 0.4 nT field observed at V1. The V2 magnetic field was predicted to be stronger than that at V1 to account for the difference in the TS locations.

### The Plasma Boundary Region

**Fig. 31:**
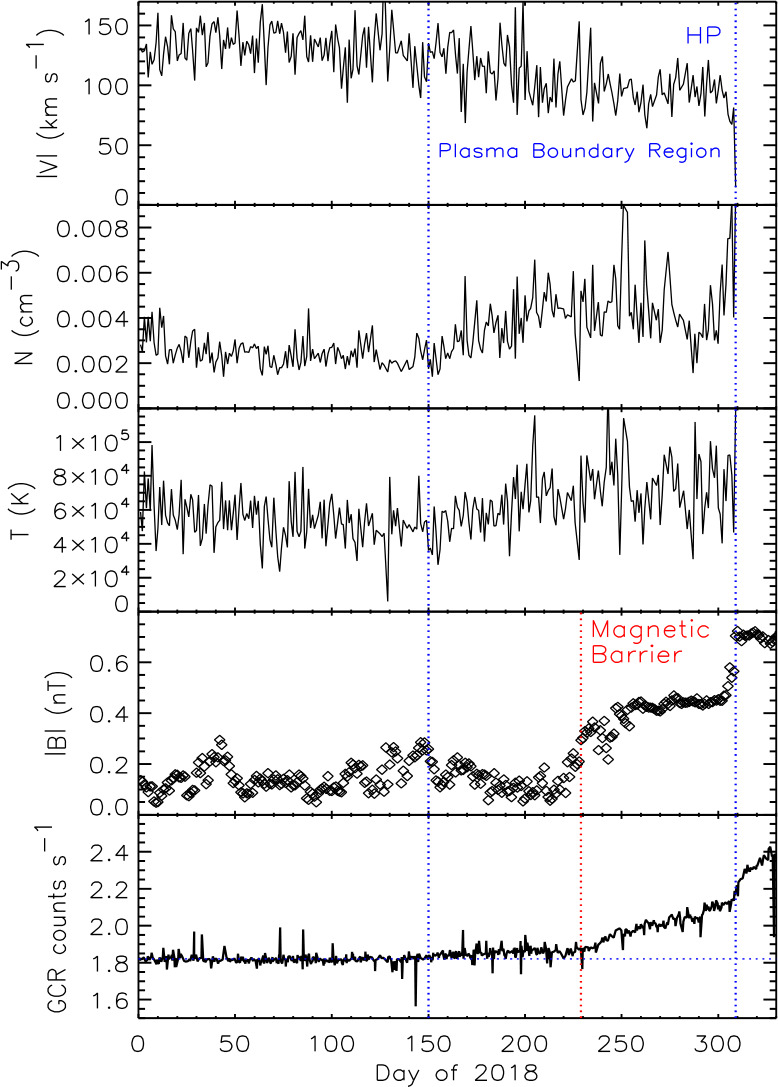
The plasma boundary layer. The panels show daily averages of the plasma speed, density, temperature, B, and GCR intensity. The dashed lines show the beginning of the plasma boundary layer, the start of the magnetic barrier, and the HP (from Richardson et al. 2019)

V2 provided the first plasma data from the HP region. Figure 31 shows a plasma boundary layer began on day 150 of 2018, 1.5 AU ahead of the HP, where the plasma speed decreases by 30%, the density increases by a factor of 2, and T increases by 30%. The LECP 53-85 keV ion intensity increase correlates well with the plasma density suggesting the whole plasma flux tube is compressed, similar to the other pressure pulses observed in the heliosheath. The GCR slope increased slightly at the beginning of this region (Fig. 31), but the magnetic field did not change at the start of the plasma boundary layer but did decrease slowly from days 150–215 as N and T increased. The origin of the plasma boundary region is not known; compression of plasma as it approaches the HP could cause an increase in the density, temperature, and LECP ion flux as observed, but would also produce an increase in B which was not observed.

### The Heliopause Transition Layer

The HP transition layer started on day 302, 2018, about 0.06 AU inside of the HP. In this boundary layer the density increases by a factor of two, the magnitudes of V_R_ and V_N_ decrease, the flow angle in the RT plane increases, and B increases by 30% (Richardson et al. 2019). This region contains solar wind plasma that is modified by the HP boundary. The V2 HP was a sharp boundary where the outward plasma flux dropped to background levels, B increased, the GCR intensity increased, and the heliospheric particle intensity decreased in the 8-hour data gap on day 309.

### Heliopause Leakage

**Fig. 32:**
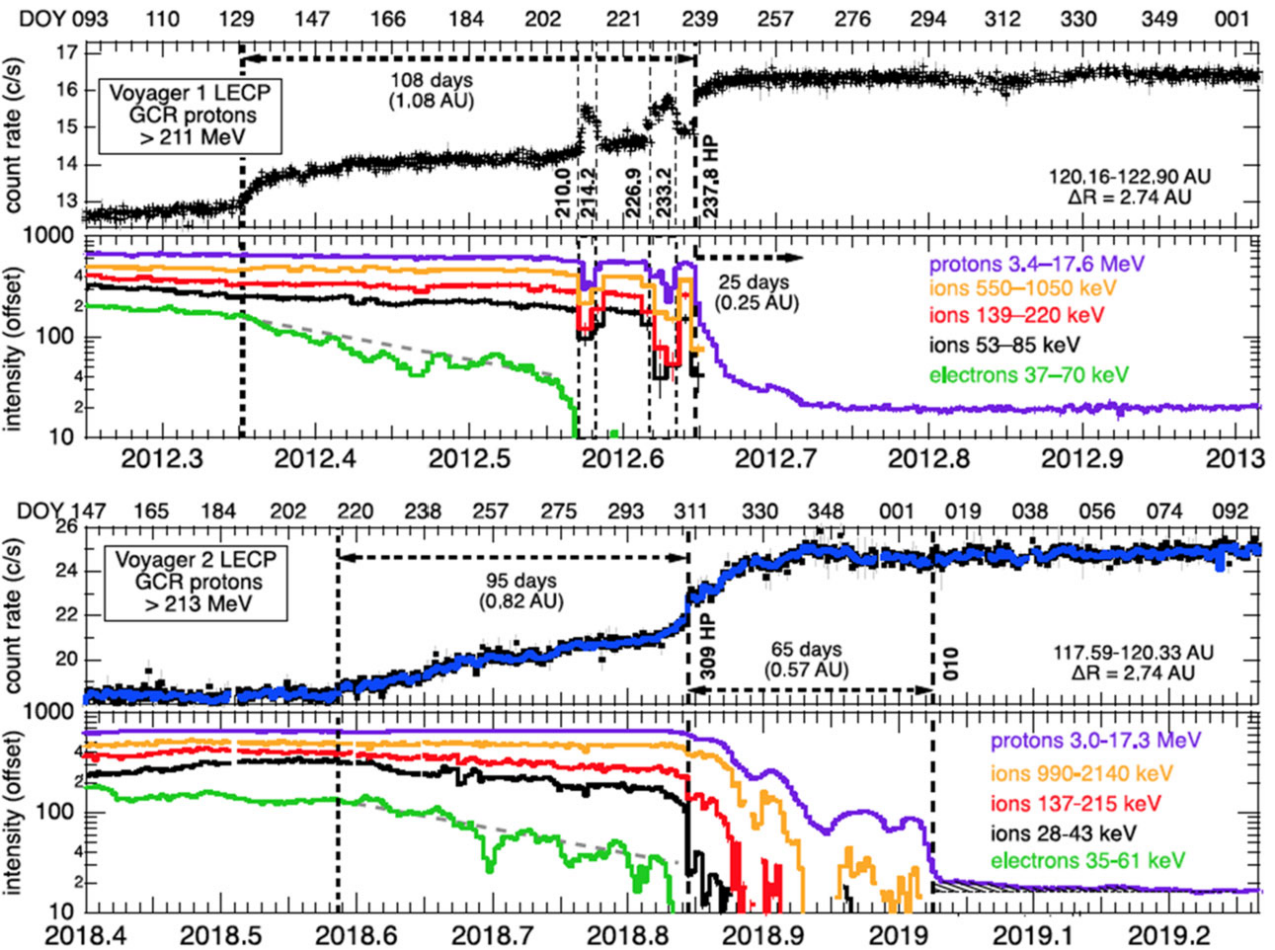
Particle intensities near the V1 (top) and V2 (bottom) HP crossings (Krimigis et al. 2019)

Figure 32 shows the particle intensity profiles across the HP (Krimigis et al. 2019). At V1 several HP precursors were observed which looked like partial HP crossings or encounters with flux tubes from the VLISM moving into the heliosheath. After the V1 HP, ACRs were observed in the VLISM for about 25 days. At V2 the leakage from the heliosheath into the VLISM was much more pronounced, especially at higher energies. A drop off in ACRs occurred 65 days after the HP but these ions did not drop to background levels until about 120 days after the HP. The GCRs at V1 very quickly approached their asymptotic value, but at V2 the GCR increase took about 30 days. The region where magnetic field lines are connected outside the HP was much larger at V2 than V1, perhaps because V2 was further from the nose of the heliosphere.

### The Heliopause

The V1 HP crossing was marked by an increase in the magnetic field strength, a decrease of heliosheath energetic particle intensities, and an increase in the GCR counting rate. After the HP, B remained high and steady, the heliosheath particles disappeared, and the GCR intensities plateaued. However, the direction of the magnetic field did not change. PWS data confirmed that this boundary was the HP, observing the higher densities, ∼0.06 cm^−3^, expected in the VLISM (Gurnett et al. 2013). Several HP precursors were observed at V1, with smaller decreases in B and the energetic particles and increases in the GCRs centered on days 212 and 230 of 2012 (Stone et al. 2013; Krimigis et al. 2013; Burlaga et al. 2013). The precursors may be flux tubes moving from the VLISM into the heliosheath.

The V2 crossing in Fig. 30 did not have precursors like those at V1. On day 309 of 2018, B sharply increased, the heliosheath energetic particle intensity decreased, the GCR counting rate increased, and the radially outward plasma currents dropped to background levels. PWS observations from days 35–55 of 2019 confirmed the density had increased to 0.04 cm^−3^, consistent with V2 having crossed into the LISM (Gurnett and Kurth 2019). The biggest surprise of the V1 HP crossing was that the direction of B did not change (Burlaga et al. 2013; Burlaga and Ness 2014). At V1 the magnetic field direction near the HP was nearly constant but different from the Parker field direction of 270° by about 20° in azimuth and 18° in elevation angle. Models disagreed on whether this lack of B rotation was a coincidence of the geometry where the HP was crossed or if the rotation of the VLISM B toward the Parker spiral direction were an intrinsic HP feature. This issue was resolved (Fig. 29) when B did not change direction at the V2 HP crossing and thus this lack of a rotation in B seems a usual, but not understood, HP characteristic (Burlaga et al. 2019).

At V2, the magnetic field azimuthal angle was very close to 270° and the elevation angle was about 20°. The strong uniform magnetic fields, the absence of energetic particles, the plateau of the GCRs and the cessatiopn of radial flow were strong evidence that V2 had crossed the HP. The lack of field rotation is still a puzzle and discussed in the modeling chapter (Kleimann et al. 2022).

## The LISM

### The LISM Temperature

The PLS instrument normally observes LISM currents in only one energy channel in one detector. This gives one observation but three unknowns, the plasma density, temperature, and speed into the cup. However, there are two times PLS can estimate the plasma temperature; when PWS measures the density and when the spacecraft rolls, giving data from different look directions. When the density is known from PWS, the measured currents in the 10-30 eV ion detector limit T and V to a 2D box in TV space. Using a reasonable range for V, T is estimated to be 30,000-50,000 K. When the spacecraft rolls PLS (sometimes) observes the variation of the currents with look angle. Fitting the currents vs. angle gives T, V, and N; the fit T is again on the order of 35,000 K (Richardson et al. 2019; 2020). The temperatures are on the upper end of those expected from MHD models (Zank et al. 1996) and may suggest heating by reconnection in the PDL (Fuselier and Cairns 2017).

### The Electron Density in the VLISM

**Fig. 33:**
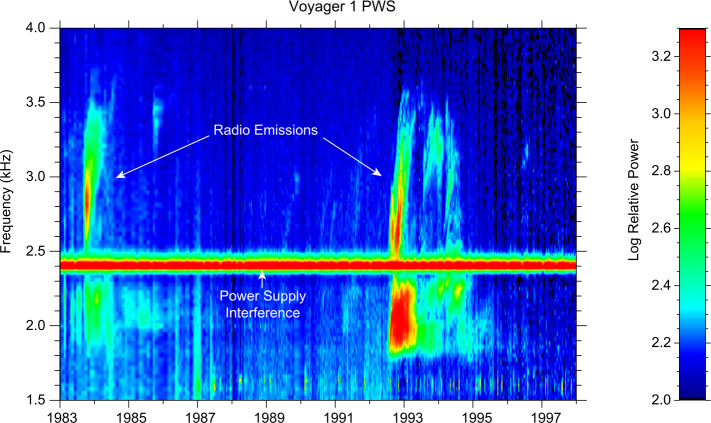
Radio emissions were observed by both Voyager PWS instrument some time after the Saturn encounters. They consist of a low-frequency component (< 2.4 kHz) that shows little frequency drift and a series of higher frequency emissions that drift in frequency with time. Gurnett et al. (1993) developed the basic explanation of these emissions as radio waves mode converted from electron plasma oscillations at the electron plasma frequency in the source. Further, they argued for the source plasma density to be high enough to explain the frequencies of the emissions, the source had to be beyond the heliopause where a strong increase of density would be expected to balance the pressure from the much lower density and hot plasma in the heliosheath

The Voyager PWS instruments began making remote measurements of the density of the VLISM as early as 1983 through the detection of radio emissions as shown in Fig. 33 in the frequency range of 1.8 to 3.5 kHz from the vantage point of heliocentric radial distances of greater than about 13 AU (Kurth et al. 1984, 1987). At the time of these measurements, the nature of the source was not understood, although sources at or beyond the heliopause were hypothesized. The basic explanation for these emissions was formulated by Gurnett et al. (1993) as a second group of radio emissions was detected beginning in 1992. Both the events starting in 1983 and in 1992 showed similar structures, a low-frequency (<2.4 kHz) component with little drift in frequency and a higher frequency component displaying frequency drifts of order 1 kHz/yr. Another, significantly weaker event commenced in 2002. The temporal spacing of the events was similar to the 11-year solar cycle, which implied a solar influence in the occurrence of the emissions. The 1983 (1992) event was first detected within 412 (408) days of a very strong Forbush decrease. A Forbush decrease is a decrease in the flux of galactic cosmic rays at Earth caused by disturbances in the interplanetary magnetic field that impede the access of the cosmic rays to the inner heliosphere. This correlation led to the hypothesis that transients from the Sun propagate through the heliosphere, form GMIRs which hit the HP and drive shocks that propagation into the VLISM. Using observed shock speeds from in situ observations of transients at a range of heliocentric distances by Ulysses, Pioneers 10 and 11, and the Voyagers, and some simple assumptions about the thickness of the heliosheath and propagation speeds therein, a time-of-flight calculation gave an estimate for the distance to the heliopause of 116 to 177 AU (Gurnett et al. 1993). This estimate encompasses the distance of the observed heliopause crossings in the range of 120 AU by both Voyagers.

**Fig. 34:**
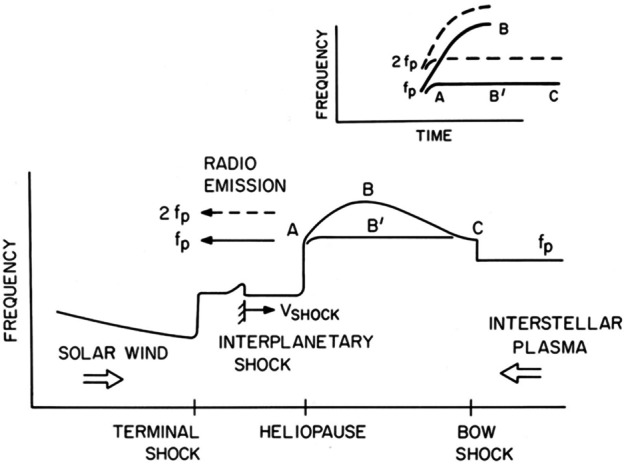
A model by Gurnett et al. (1993) to explain the frequency drift of the radio emissions detected by Voyager inside the heliosphere. The propagation of a shock through a density gradient beyond the heliopause causes electron plasma oscillations at the plasma frequency that mode couple into radio emissions and propagate into the heliosphere, where they are detected by the Voyagers

**Fig. 35:**
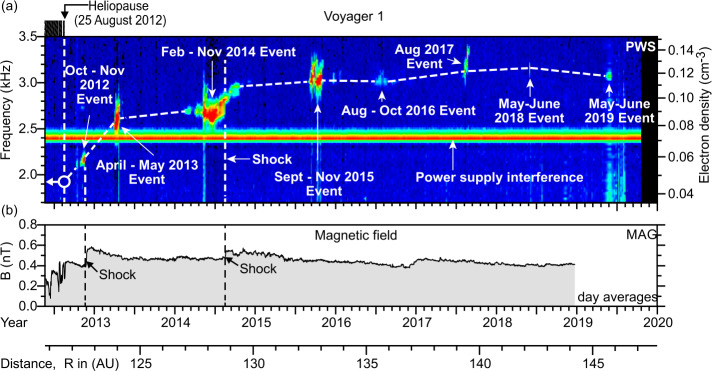
Detections of electron plasma oscillations by Voyager 1 after it crossed the heliopause. The increasing frequency of the events with increasing time and radial distance shows a radial gradient in the VLISM (Gurnett and Kurth 2019). These plasma waves were predicted to be present in the VLISM in the early 1980’s and are responsible for the observed radio emissions

Gurnett et al. (1993) explain the frequency drift of the radio emissions as owing to the propagation of the shocks through a density gradient beyond the heliopause (Fig. 34). The shocks accelerate electron beams which excite electron plasma oscillations at the local electron plasma frequency. The plasma oscillations then mode-convert into radio emissions that propagate freely away from the source. These radio waves were detected by Voyager outside 10 AU. Assuming the primary radio emission is at the electron plasma frequency $f_{pe}$, the frequency of the radio emission gives the plasma frequency at the source. Hence, the frequency drift is a result of shocks propagating through the VLISM. With $n_{e}$ related to the electron plasma frequency (Hz) by $n_{e} = (f_{pe}/8980)^{2}$, the VLISM electron densities at the radio sources range from about 0.04 cm^−3^ to 0.15 cm^−3^. Voyager PWS measurements in the VLISM substantiated the earlier interpretations of the kHz radio observations. The Voyagers were at the right time and location to observe electron plasma oscillations driven by shocks propagating into the VLISM from solar transients. As of 2022, eight intense electron plasma oscillations events have been observed by Voyager 1 as shown in Fig. 35 (Gurnett and Kurth 2019). The frequencies of these emissions show that the VLISM electron density increases from 0.04 cm^−3^ near the heliopause to 0.12 cm^−3^ 20 AU beyond the heliopause. Electron plasma oscillations are associated with an electron foreshock not unlike those ahead of planetary bow shocks in the solar wind; however, these shocks are associated with solar transients that are transmitted through the heliopause and propagate through the VLISM (Gurnett et al. 2015). These have been observed at a rate loosely averaging once per year.

**Fig. 36:**
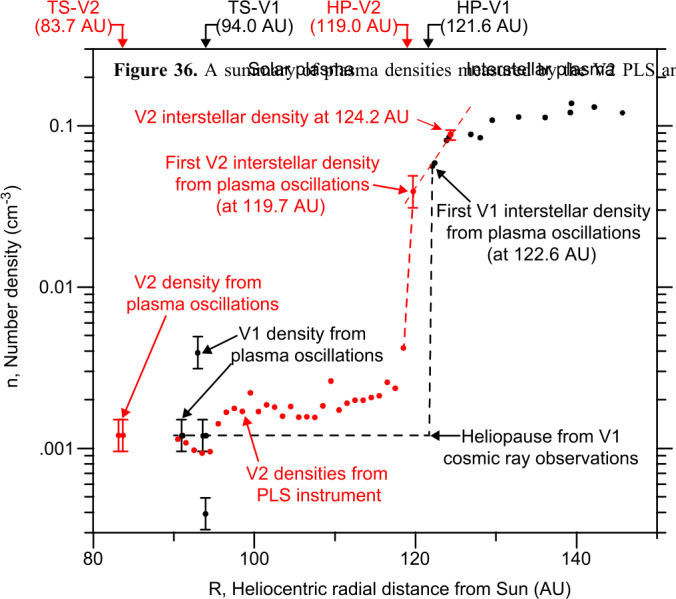
A summary of plasma densities measured by the V2 PLS and the V1 and V2 PWS instruments beginning just inside the termination shock. The two plasma oscillation events detected by V2 after it crossed the heliopause show a radial density gradient remarkably similar to that observed by V1. Given that the two Voyagers are separated by 70° as viewed from the sun, the radial gradient is a large-scale feature of the VLISM, at least toward the nose of the heliosphere (Kurth and Gurnett 2020)

The V2 PWS has detected two plasma oscillation events since it crossed the heliopause in 2018. The V2 instrument no longer produces high resolution wideband data, but the PWS spectrum analyzer channels allow determination of the VLISM plasma density (Kurth and Gurnett 2020). The two V2 measurements are plotted with PLS and PWS measurements from upstream of the termination shock up to beyond 146 AU in Fig. 36. A clear radial density increase is observed by both Voyagers despite the broad separation in their locations.

**Fig. 37:**
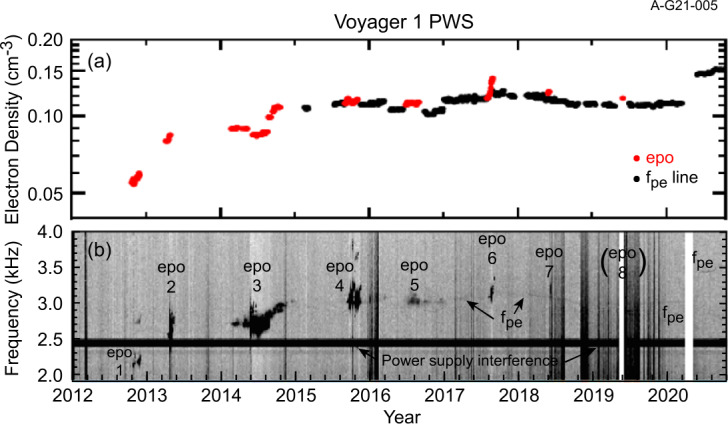
The detection of a very weak line at the electron plasma frequency starting in 2015 (**b**) (from Burlaga et al. 2021b) and the associated electron density profile from 2012 into late 2021 (**a**). The intense emissions labeled ‘epo’ are the electron plasma oscillation events shown in Fig. 3; the densities inferred from their frequencies are shown in red in panel (**a**). The black points in panel (**a**) are densities inferred from the weak thermal plasma line appearing during after 2015 panel (**b**)

Figure 37 shows that starting in 2015 a very weak line has been observed in the V1 PWS wideband observations at the electron plasma frequency that is likely due to thermal plasma oscillations (Burlaga et al. 2021b; Ocker et al. 2021). The densities obtained from the plasma oscillation events and the thermal plasma line are given in Fig. 36. As noted by Burlaga et al. (2021b), the plasma densities from the thermal plasma line show density increases at two pressure fronts recognized in the Voyager 1 magnetometer data. The ratios of the plasma densities and the magnetic fields before and after the shock in 2014 and at both pressure fronts are remarkably similar.

### The Propagation of Solar Transients into the VLISM

As discussed above, solar transients are the primary drivers of the plasma oscillations observed in the VLISM. CMEs and CIRs are the two primary types of solar transients. Note that the propagation time of a solar transient from the Sun to the heliopause is several hundred days, during which many solar transients can occur. Therefore, interactions between the solar transients during the transit time and formation of an MIR occur in the outer heliosphere. A radio wave event observed by Voyager in the VLISM is most likely caused by merging of multiple transients.

MIRs in the outer heliosphere can form from the merging of a series of ICMEs (e.g., Burlaga 1995; Wang and Richardson 2002; Richardson et al. 2002). The Voyager measurements in the VLISM enable timing analysis of the propagation of solar transients into the VLISM. The radio and shock events observed at Voyager 1, as shown in Fig. 34, also allow investigations of the ultimate destiny of solar transients as they approach the outer heliosphere and how they affect the VLISM.

**Fig. 38:**
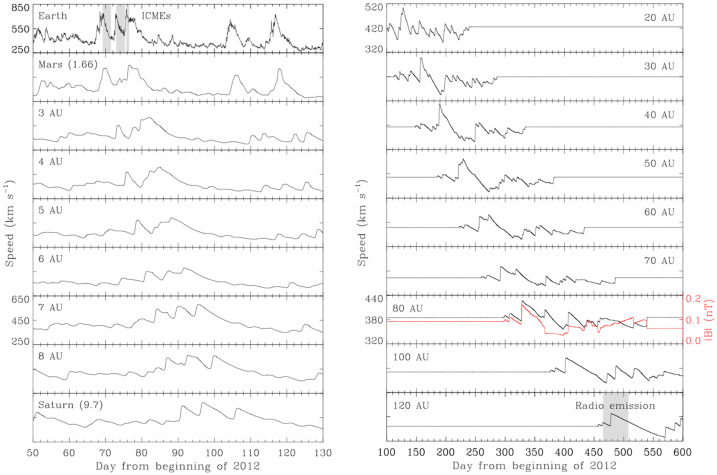
MHD propagation of solar wind streams from the Earth to 120 AU. The upper left panel shows the observed solar speed at Wind, and the other panels show the predicted speeds at various distances. The shaded regions in the first panel represent the ICME intervals at the Earth, and the shaded region in the last panel indicates the period of the radio emission observed by Voyager 1. The predicted magnetic field is plotted at 80 AU to show the size of the MIR. Reproduced from Liu et al. (2014)

The April-May 2013 radio wave event in Fig. 35 helped determine that Voyager 1 had crossed the heliopause, providing the first evidence that the density had increased to the expected VLISM levels. This event is hypothesized to have been produced by the 2012 March CMEs hitting the heliopause (Gurnett et al. 2013). Liu et al. (2014) made the first attempt to establish the timing of the propagation of solar transients into the VLISM, combining wide-angle imaging observations from STEREO, in situ measurements, and MHD propagation of the measured solar wind disturbances. In 2012 March, the active region NOAA AR 11429, one of the biggest active regions in solar cycle 24, exhibited extraordinary activity (Liu et al. 2014). The active region emitted a series of large CMEs as it rotated with the Sun from the east to west. A cluster of shocks and ICMEs were observed near the Earth, and their propagation outward was predicted using an MHD model (see Fig. 38). The transient stream interaction results in the formation of a large MIR preceded by a shock in the outer heliosphere. The predicted arrival time of the shock and MIR at 120 AU is April 22 2013, which agrees with the April-May 2013 radio emission period and the time of a transient disturbance in galactic cosmic rays (Gurnett et al. 2013; Krimigis et al. 2013).

This MHD model does not include the transition across the termination shock and the heliosheath. Liu et al. (2014) use passage of a shock through the Earth’s bow shock and magnetosheath as an analogy, to determine the speed of the shock in the heliosheath. Subsequent MHD simulations are fully three-dimensional (3D) and multi-fluid (e.g., Fermo et al. 2015; Kim et al. 2017; Guo et al. 2021). These recent models have the advantage of including the termination shock, heliosheath and heliopause, but face difficulties such as how to determine the 3D solar wind input, and reproduce correct locations of the heliospheric structures and precise timing with the Voyager 1 measurements. Nevertheless, Kim et al. (2017) reproduce some of the shocks in the magnetic field observed at Voyager 1, assuming an ad hoc 3D solar wind input with a prescribed polar coronal hole geometry. Their results indicate that merging of CIRs may also have played a role in the formation of some of the MIR shocks.

**Fig. 39:**
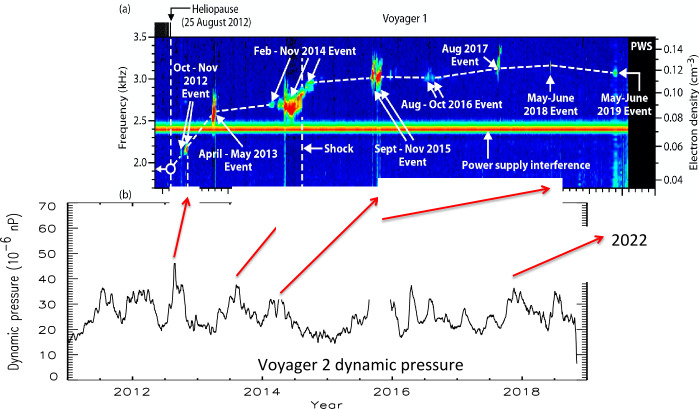
Top: the plasma oscillations and shocks observed at V1. Bottom: the dynamic pressure observed by V2 in the heliosheath. The red lines show the time that each V2 pressure peak is predicted to arrive at V1

Observationally, the timing of events at V1 and V2 can also be compared (Richardson et al. 2017). V1 crossed the heliopause into the VLISM in 2012 when Voyager 2 was still in the heliosheath. From 2012.5–2016.5, solar maximum conditions persisted in the heliosheath with five MIRs observed at Voyager 2. These MIRs occur at a similar frequency to the transients observed in the VLISM by Voyager 1. Figure 39 shows that the timing between Voyager 1 and 2 measurements indicates that the transients observed in the VLISM by Voyager 1 may have been driven by the MIRs observed at Voyager 2.

## Summary

The Voyager spacecraft provided the first survey of the heliosphere from the Earth into the LISM, making the first measurements of the outer heliosphere where PUIs dominate the thermal pressure, of the termination shock, the heliosheath, heliopause, and the VLISM. These observations provided many surprises and have left us with many puzzles for future missions to answer. The Voyager spacecraft have sufficient power to operate all instruments until the mid-2020s; after this time, the instruments will be turned off serially which will extend the useful life of the spacecraft to at least 2030. New Horizons is providing another view of the outer heliosphere and may cross the TS and enter the heliosheath, making the first direct measurements of the PUIs. IBEX (and soon IMAP) provide a global view of the heliosphere through ENA measurements. But more missions are needed!
